# Selective Allosteric Inhibition of MMP9 Is Efficacious in Preclinical Models of Ulcerative Colitis and Colorectal Cancer

**DOI:** 10.1371/journal.pone.0127063

**Published:** 2015-05-11

**Authors:** Derek C. Marshall, Susan K. Lyman, Scott McCauley, Maria Kovalenko, Rhyannon Spangler, Chian Liu, Michael Lee, Christopher O’Sullivan, Vivian Barry-Hamilton, Haben Ghermazien, Amanda Mikels-Vigdal, Carlos A. Garcia, Brett Jorgensen, Arleene C. Velayo, Ruth Wang, Joanne I. Adamkewicz, Victoria Smith

**Affiliations:** 1 Department of Biology, Gilead Sciences, Inc., Foster City, California, United States of America; 2 Department of Process Development, Gilead Sciences, Inc., Oceanside, California, United States of America; CWRU/UH Digestive Health Institute, UNITED STATES

## Abstract

Expression of matrix metalloproteinase 9 (MMP9) is elevated in a variety of inflammatory and oncology indications, including ulcerative colitis and colorectal cancer. MMP9 is a downstream effector and an upstream mediator of pathways involved in growth and inflammation, and has long been viewed as a promising therapeutic target. However, previous efforts to target matrix metalloproteinases (MMPs), including MMP9, have utilized broad-spectrum or semi-selective inhibitors. While some of these drugs showed signs of efficacy in patients, all MMP-targeted inhibitors have been hampered by dose-limiting toxicity or insufficient clinical benefit, likely due to their lack of specificity. Here, we show that selective inhibition of MMP9 did not induce musculoskeletal syndrome (a characteristic toxicity of pan-MMP inhibitors) in a rat model, but did reduce disease severity in a dextran sodium sulfate-induced mouse model of ulcerative colitis. We also found that MMP9 inhibition decreased tumor growth and metastases incidence in a surgical orthotopic xenograft model of colorectal carcinoma, and that inhibition of either tumor- or stroma-derived MMP9 was sufficient to reduce primary tumor growth. Collectively, these data suggest that selective MMP9 inhibition is a promising therapeutic strategy for treatment of inflammatory and oncology indications in which MMP9 is upregulated and is associated with disease pathology, such as ulcerative colitis and colorectal cancer. In addition, we report the development of a potent and highly selective allosteric MMP9 inhibitor, the humanized monoclonal antibody GS-5745, which can be used to evaluate the therapeutic potential of MMP9 inhibition in patients.

## Introduction

Matrix metalloproteinase (MMP)-mediated proteolysis plays a key role in modulation of cellular homeostasis: MMPs can initiate, amplify, or downregulate signaling cascades involved in growth and inflammation by activating cytokines and liberating sequestered growth factors, and can modify tissue architecture by degrading structural components of the extracellular matrix (ECM) [1–6]. Of the 23 MMP family members, MMP9 (also known as gelatinase B) shows particular promise as a therapeutic target, given the body of evidence demonstrating its participation in pathological processes that contribute to chronic inflammation, tumorigenesis, and metastasis [5–7].

Dysregulated MMP9 expression and activity are associated with several inflammatory disorders, including ulcerative colitis (UC) [1, 7–12]. UC is a relapsing/remitting autoimmune inflammation of the colon [13–16] that features induction of MMP9 protein levels and proteolytic activity in areas of active disease [10, 11, 17]. MMP9 activity in UC is implicated in both generation and perpetuation of an inflammatory state—it is induced by pro-inflammatory cytokines such as TNF-α and IL1- α [18–20] and it can help sustain pro-inflammatory processes by releasing TNF-α and TGF-β, by potentiating IL-8, and by activating IL1-β [4, 21–26]. MMP9 also can contribute to the inflammatory milieu through proteolysis of the basement membrane (BM) constituents collagen IV and laminin [7]. Destruction of epithelial BM, a defining feature of UC [13, 14, 16, 18], can result in epithelial cell apoptosis [27], which contributes to the loss of integrity of the colonic mucosal epithelial barrier, further exacerbating inflammation. Similarly, disruption of the endothelial BM can facilitate lymphocyte and neutrophil transmigration to the site of inflammation [28–30].

Chronic UC andMMP9 expression in UC are risk factors for the development of colorectal carcinoma (CRC) [15, 31–33], and although the exact path from chronic inflammation to dysplasia to neoplasm is not clear, the involvement of MMP9 in processes that enable the establishment and propagation of both of these diseases [1, 6, 7, 34, 35] suggests that it may play a role in the progression of UC to cancer. MMP9 expression is elevated and is correlated with poor prognosis in a wide array of tumors, including CRC [5, 6, 35–47], and it plays multiple roles in the process of tumorigenesis: MMP9 is produced by tumor cells as well as by stromal inflammatory cells such as tumor-associated macrophages (TAMs) and neutrophils, and is a key mediator of the tumor-stroma crosstalk that results in reciprocal activation of pro-oncogenic signaling in these two compartments [48–52]. MMP9 promotes metastasis by facilitating tumor cell migration and invasion via cleavage of BM and other ECM components [53], and it has also been implicated in primary tumor growth by virtue of its position as both a downstream target [54–63] and an upstream regulator of key oncogenic signaling pathways. In the latter capacity, MMP9 may enable pro-oncogenic signaling via its ability to liberate growth factors such as EGF, FGF-2, and VEGF [64–67], and to modulate integrin and receptor tyrosine kinase function [54, 68, 69]. Ultimately, these different aspects of MMP9 function work in concert to effect the signaling dysregulation and matrix proteolysis that contribute to the growth and spread of tumors [53, 64, 70–73].

The relevance of MMP9 in the pathology of certain inflammatory and oncology indications has been demonstrated by reports showing that *mmp9-/-* mice exhibited decreased disease severity in preclinical models of colitis and rheumatoid arthritis, and also displayed reduced tumor growth and/or reduced metastases in several cancer models [1, 66, 74–81]. Although these and other published observations suggest that MMP9 is a compelling therapeutic target, previous efforts to target MMPs (including MMP9) utilized pan-specific or semi-selective inhibitors, and were unsuccessful due to dose-limiting side effects such as musculoskeletal syndrome (MSS) and/or to a general lack of clinical benefit [1, 17, 39, 82–84]. In retrospect, the lack of a therapeutic window with these broader-spectrum MMP inhibitors is understandable, given the roles that MMPs can play in critical homeostatic processes [22, 85].

Here, we report the development of a highly selective and potent allosteric antibody inhibitor of MMP9: we show that inhibition of MMP9 is efficacious in mouse models of UC and colorectal cancer, and that this therapy does not induce MSS in a rat model. We propose that selective inhibition of MMP9-mediated pathological signaling and matrix proteolysis is a novel therapeutic opportunity in inflammatory conditions such as UC, and in cancers such as CRC.

## Materials and Methods

### Tissues

Fresh-frozen (FF) and formalin fixed and paraffin embedded (FFPE) human tissues were obtained from Cureline, Inc. (Burlingame, CA), Asterand (Detroit, MI), or Folio Biosciences (Powell, OH).

### Recombinant MMP9 proteins and immunization

Human MMP9 protein was generated by cloning the full length cDNA into the pSecTag2hygro (B) vector (Life Technologies, Carlsbad, CA) and transiently transfecting it into HEK293 cells (ATCC, Manassas, VA). The conditioned medium was purified with a Ni-Sepharose Fast Flow 16/20 XK column (GE Life Sciences, Pittsburgh, PA). This protein and Ribi adjuvant were used to immunize BALB/c mice (Jackson Laboratories, Bar Harbor, Maine) via the foot pad. Mice with serum antibody titers against MMP9 were used to make hybridoma libraries via the fusion of B-cells isolated from lymph nodes. These libraries were subcloned by single cell sorting to generate clonal populations from which anti-human MMP9 antibody AB0041 was identified. The immunizations, hybridoma library creation, and antibody cloning were conducted at Antibody Solutions (Sunnyvale, CA). The anti-mouse MMP9 monoclonal antibody AB0046 was similarly generated, with the exceptions that MMP9 knockout mice (Jackson Laboratories) were used and that a mouse MMP9 protein composed of the pro and catalytic domains only (aa 1–445) was used for immunization. For specificity analysis, full length MMP family proteins were purchased from R&D Systems (Minneapolis, MN).

### AB0041 and AB0046 antibody production and purification

Hybridoma cells expressing AB0046 or AB0041 were cultured in IMEM, 10% Fetal Bovine Serum (low IgG), penicillin/streptomycin (1X), 5% Hybridoma Cloning Factor, and HT media supplement (1X) (Life Technologies, Grand Island, NY). Ascites fluid was generated, was purified by batch mode on MabSelect SuRe resin (GE Healthcare, Piscataway, NJ), and was formulated in phosphate-buffered saline (PBS; 10 mM sodium phosphate, 140 mM sodium chloride). Antibody purity was assessed by resolving reduced and non-reduced samples with SDS-PAGE 4–12% Bis-Tris gels and staining with Simple Blue Safe stain (Invitrogen). The purified antibodies were shown to contain less than 5% aggregates by SEC-HPLC using TSKgel G3000SWxl column from Tosoh (King of Prussia, PA). LAL testing (Endosafe, Charles River Laboratories, Charleston, SC) was used to confirm antibody preparations contained less than 5 EU/mg endotoxin.

### MMP9 direct binding enzyme-linked immunosorbent assay (ELISA)

Direct binding antigen-down ELISA assays with purified recombinant MMP9 proteins were developed to measure the apparent binding affinity of AB0046, AB0041, and GS-5745. All washes were with PBS + 0.05% Tween-20 (PBST). Full-length MMP9 proteins were coated onto Maxisorp plates (NUNC, Rochester, NY), followed by blocking with PBS + 5% (w/v) bovine serum albumin (BSA; EMD Millipore). Plates were then washed and serial dilutions of anti-MMP9 antibodies in PBS were added. After washing, plates were incubated with horseradish peroxidase (HRP)-conjugated secondary antibody (Thermo Scientific, Fair Lawn, NJ) diluted 1:10,000 in PBS + 0.5% (w/v) BSA then washed and developed using 3,3’,5,5’- tetramethylbenzidine (TMB; Sigma, St.Louis, MO) for 1 minute. The reaction was quenched by the addition of 1 M hydrochloric acid. Quantification was carried out on a SpectraMax M5 plate reader (Molecular Devices, Sunnyvale, CA) in absorption mode at a wavelength of 450 nm. Apparent dissociation constants (K_D(ELISA)_) were determined using SoftMax Pro software (Molecular Devices) by plotting the absorbance values vs. the concentration of antibody and fitting the data to a 4-parameter logistic equation, with the “C” parameter equation defined as the apparent K_D_.

### Epitope mapping of AB0041 and AB0046

All mutant MMP9 constructs used in epitope mapping were generated by mutating mouse and human MMP9 expression vectors using a QuikChange II Site Directed Mutagenesis Kit (Stratagene, La Jolla, CA). Individual clones were verified by DNA sequence analysis, and mutant MMP9 proteins were generated by transient transfection in HEK293 cells. Conditioned medium was harvested after 24 to 48 hours and used to coat a His-Select Hi-Capacity Ni2^+^ coated plate (Sigma), overnight at 4°C. The following day plates were washed in PBST and blocked with 5% BSA in PBS for one to two hours at ambient temperature, followed by incubation with either 10 nM or 1 nM antibody diluted in PBST. Plates were washed and incubated with a goat-anti-mouse IgG- HRP-conjugated secondary antibody (Jackson ImmunoResearch, West Grove, PA,) diluted 1:10,000 in 0.5% BSA in PBS. Plates were washed, developed, and read as described above.

### MMP9 activity assays

MMP9 activity was assessed using quenched fluorogenic substrates; cleavage generates fluorescence that is proportional to the amount of enzyme activity [86]. Human and mouse assays used QXL 520-γ-Abu-P-Cha-Abu-Smc-HA-Dab(5-FAM)-AL-NH2, where Smc = S-methyl- L-cysteine, Abu = 2-aminobutyric acid and Cha = β-cyclohexylalanine (AnaSpec Inc., Fremont, CA), and the rat assay used Mca-PLGL-Dpa AR-NH2; (R&D Systems, Minneapolis, MN). Purified recombinant MMP9 from HEK293 cells was activated by overnight incubation at 37°C with 4-aminophenylmercuric acetate (APMA) in 50 mM Tris pH 7.5, 10 mM calcium chloride, 150 mM sodium chloride and 0.05% Brij-35 buffer [87]. MMP9 protein (63 pM human, 125–150 pM mouse, or 1 nM rat) was transferred to a 96-well black plate (Costar) and mixed with serially diluted antibody. After MMP9-antibody complex formation, substrate (20 μM for human and mouse assays, 10 uM for rat) was added and fluorescence was monitored in kinetic mode at 37°C on either a SpectraMax M2 or M5 plate reader (excitation 320 nm, emission 405 nm) or an Infinite M1000 plate reader (Tecan, Switzerland) using an excitation wavelength of 494 nm and an emission wavelength of 521 nm. The slope of the curve (relative fluorescence units [RFU] per minute) was determined in the linear region, and then plotted against concentration of the antibody using a 4-parameter curve fitting algorithm to determine an IC_50_ value. To determine the mode of MMP9 inhibition, the activity assay was conducted as described above using four different concentrations of the labeled QXL-FAM peptide substrate.

### DQ-collagen IV and DQ-gelatin assays

Human DQ-collagen IV and porcine DQ-gelatin (Life Technologies) are quenched fluorescein-conjugated proteins that fluoresce upon digestion. Human or mouse full length recombinant MMP9 proteins were activated as described above. Human MMP9 at 1 nM or 0.25 nM (collagen IV or gelatin assay, respectively) or mouse MMP9 at 1 nM was added to a 96-well black microplate containing serial dilutions of antibody. After MMP9-antibody complex formation, 25 ug/ml substrate was added and fluorescence was monitored on a SpectraMax M5 plate reader (Molecular Devices) set at 37°C with excitation/emission wavelengths of 495/515 nm for 3 hours (collagen IV) or on a PHERAstar FS plate reader (BMG LAbtech) with excitation/emission wavelengths of 485/520 nm for 30 minutes (gelatin). Data were corrected by subtraction of their respective negative (no enzyme) control wells, and relative fluorescence units (RFU) were converted to percent inhibition, which was then plotted against concentration of the antibody; a 4-parameter curve fit was used to determine an IC_50_.

### TNF-α fusion protein cleavage assay

The recombinant human pro-TNF-α fusion protein cleavage assay (R&D Systems, 1012-PS-010) was performed following the manufacturer’s protocol. Active human recombinant MMP9 (Millipore, PF024) or ADAM17 (R&D Systems, 903-ADB) was mixed with 10 uM AB0041 or 10 uM BB-94 (Selleck Chemicals, S7155) and incubated for 30 minutes at room temperature. TNF-α fusion protein substrate was then added and incubated overnight at 37°C. Western blot analysis was performed with a rabbit polyclonal TNF-α antibody (Cell Signaling, 3707). Recombinant human TNF-α (R&D Systems, 201-TA) was used as a positive control.

### Rat MSS model

Thirty male Lewis rats were obtained from Harlan laboratories (Livermore, CA) and were acclimatized for five to seven days prior to initiation of the study. Rats were randomized into 5 groups (3 control; 2 experimental) of 6 rats/group based on their body weight. Rats were housed in individual cages in a temperature-controlled room with a 12-hour light/dark cycle, and had ad libitum access to drinking water and animal chow throughout the course of the study. AB0041 (50 mg/kg) or vehicle (PBS pH 6.5, 0.01% Tween-20) was administered to 6 rats twice weekly via intravenous tail vein injection. As a positive control for MSS, 6 rats were treated with marimastat (Santa Cruz Biotechnology, Santa Cruz, CA) through a surgically implanted subQ Alzet pump (Alzet, Cupertino, CA). Each pump contained a total of 60 mg marimastat, which was delivered at a rate of 2.5 μl/hour for a period of 28 days. A fourth group of 6 rats received the vehicle used for marimastat dilution (50% DMSO/50% water) through a SubQ Alzet pump. Marimastat release rate was between 6.8 mg/kg/day (at study initiation) and 5.7 mg/kg/day (at study termination). Animals were observed and scored daily for MSS symptoms according to the criteria cited in Renkiewicz et al. [88]. Resting posture, gait and willingness to move: resting posture was scored as 0 (normal), 1 (resting on one foot) or 2 (resting on neither one foot nor two feet). Gait was scored as either 0 (normal), 1 (avoids use of one hind foot) or 2 (avoids use of both hind feet). Willingness to move upon stimulation was scored as either 0 (normal movement), 1 (somewhat reluctant to move), 2 (moderately reluctant to move) or 3 (very reluctant to move). At study termination (day 28), limbs were harvested and fixed in 10% neutral buffered formalin for histopathologic analysis. Limbs were decalcified and then trimmed, processed, embedded in paraffin, sectioned, stained with hematoxylin and eosin (H&E) and examined microscopically. The MSS study was conducted at Aragen Biosciences Inc. (Morgan Hill, CA).

### DSS-induced colitis model

Seventy-five Male C57BL/6 mice were obtained from Charles River Laboratories (Wilmington, MA) and were acclimatized for 5 days prior to study commencement and monitored to confirm health. The study was performed in animal rooms provided with HEPA filtered air at a temperature of 70°F +/-5°F and 50% +/- 20% relative humidity. The room was on an automatic timer for a light/dark cycle of 12 hours on and 12 hours off with no twilight. Sterile Bed-O-Cobs bedding was changed a minimum of once per week. Animals were fed with sterile Purina Labdiet 5053 rodent diet, and sterilized water was provided ad libitum.

Mice were randomized based on body weight into 5 groups of 14 animals and 1 group of 5 animals prior to the start of the study. Colitis was induced by administration of 3% w/v dextran sodium sulfate (DSS) (MP Biomedicals; MW 36,000–50,000; product no. 160110; lot no. 5237K) in the drinking water on study days 0 to 5. DSS solution was replaced with a freshly prepared solution on day 3. One group of mice (n = 5) did not receive DSS and served as the no disease control group. On study day 6, 14 animals per group were intraperitoneally administered either PBST vehicle, isotype control antibody (30 mg/kg), AB0046 (30 mg/kg), or etanercept (10 mg/kg, Clayworth Healthcare, Castro Valley, CA). Vehicle and antibodies were dosed on days 6, 9, and 12, and etanercept was dosed on days 6, 8, 10, and 12. All animals were weighed and assessed visually for the presence of diarrhea and bloody stool daily. On study termination (day 14), all animals underwent video endoscopy of the lower colon with a small animal endoscope (Karl Storz Endoskope, Germany). Animals were anesthetized with isoflurane and colitis was scored visually with the following scale [89]: 0 (for normal), 1 (for loss of vascularity), 2 (for loss of vascularity and evidence of friability), 3 (for friability and erosions), and 4 (for severe ulceration). Each mouse was assigned a single score (termed endoscopy score) that corresponded to the most severe damage observed throughout the entire length of the colon. At study termination, animals were sacrificed by exposure to C0_2_. To evaluate colitis severity histologically, a board-certified veterinary GI pathologist, blinded with respect to study groups, evaluated H&E-stained FFPE step sections of colon tissue. Five to eight separate 5 μm-thick sections taken from distinct regions of the lower 5 cm of each colon were scored for morphological changes and/or injury to the epithelium, connective tissue, and submucosa using a 4-point scale [89]. Scoring of inflammation and edema was as follows: 0 (none present), 1 (rare foci/minimal), 2 (scattered regions or mild/diffuse), 3 (numerous regions or moderate diffuse), 4 (marked). Scoring of mucosal necrosis was as follows: 0 (none), 1 (<25% affected), 2 (26–50% affected), 3 (51–75% affected), and 4 (>76% affected). These scores were averaged to obtain a single mean score per mouse per parameter. This study and the associated histopathological analysis (including selection of representative images) was conducted by Biomodels, LLC.

### HCT116 surgical orthotopic xenograft model

All three HCT116 preclinical studies were carried out by AntiCancer Inc. (San Diego, CA). Female NCr nu/nu mice were obtained from Charles River (Wilmington, MA) and were bred by AntiCancer Inc. to produce study animals. Test animals were maintained by in a HEPA-filtered environment with a 14 hour light/10 hour dark cycle for the experiment. Cages, food and bedding were autoclaved; LabDiet mouse chow was obtained from PMI Nutrition International Inc. (Brentwood, MO) and was provided ad libitum, as was drinking water. Mice were randomized based on body weight into 4 groups (one control group; 3 experimental groups) of 15 animals. Pre-implantation tumor stocks of the human colorectal cancer cell line HCT116 expressing GFP (AntiCancer Inc.) were prepared by subcutaneously injecting the HCT116-GFP cells at a concentration of 5 x 10^6^ cells /100 ul into the flank of nude mice. After expansion, tumor tissues were harvested from mice and cut into fragments of approximately 1 mm^3^. Two such tumor fragments were then surgically orthotopically implanted (SOI) adjacent to the colon of each study animal, under Ketamine/Acepromazine/Xylazine anaesthesia.

When the surgically implanted tumors reached a mean volume of approximately 70–100 mm^3^, mice were divided into treatment groups. Treatment was initiated 16 days after tumor implantation for Study 1 and Study 3, and 17 days post-implantation for Study 2. All mice treated with the AB0041/AB0046 cocktail received a loading dose of AB0046 at 50 mg/kg on the first day of treatment. AB0041 and AB0046 were thereafter dosed by intraperitoneal injection twice per week at 15 mg/kg, either singly or in combination (in the combination group, each antibody was present at 15 mg/kg). Control mice were dosed with vehicle (PBS, 0.01% Tween-20). Primary tumor sizes and body weights were measured (by caliper or by electronic scale, respectively) twice a week for Study 2 and Study 3, and once a week for Study 1. Caliper-based size estimates were obtained by measuring the perpendicular minor dimension (W) and major dimension (L) of the palpated tumor. Approximate tumor volume (mm^3^) was calculated by the formula (W^2^x L)/2.

Mice were terminated at 20 days after treatment initiation (Study 3), 18 days after treatment initiation (Study 2), or 28 days after treatment initiation (Study 1). Triggers for euthanasia were: tumor volume >2000 mm^3^, >20% weight loss, observed interference with a vital physiological function, or ulceration or necrosis of tumor. The primary colon tumor and any organs with metastasis were harvested at study end, and the primary tumor was weighed after excision. Tumors were bisected, and one half was fixed in 10% neutral buffered formalin solution for histology analysis. The other half and all GFP-positive metastatic tumors from other organs were placed in tissue cassettes and were snap-frozen in liquid nitrogen. The FluorVivo imaging system (INDEC Biosystems, Santa Clara, CA) was used for whole body imaging. At necropsy, open imaging was performed in the thoracic cavity and abdominal area for inspection of metastasis to the lymph nodes, lung and other areas. The presence of necrotic tissue in H&E-stained tumor sections was assessed by a board-certified pathologist.

### Immunohistochemistry

Frozen tissues were embedded in Optimal Cutting Temperature (OCT-Tissue Tek, VWR, Brisbane, CA) compound by immersion in an isopentane dry ice bath (-70°C). Tissues were retrieved and stored at -80°C. Tissue-containing OCT blocks were sectioned in a cryostat (Leica CM1850), and cut into 5 μm-thick cryosections, which were placed onto SuperFrost positively charged slides (VWR). For formalin-fixed paraffin-embedded (FFPE) tissue blocks, 5 μm-thick sections were cut, mounted on SuperFrost slides, and baked at 60°C for approximately 20 minutes.

Unless otherwise stated, all IHC reagents and equipment were from Biocare Medical (Concord, CA), IHC procedures were performed at room temperature, and all slides were stained using the Nemesis 3600 from Biocare Medical. Pre-fixing (frozen tissue sections): slides were fixed with 4% paraformaldehyde (VWR) and then rinsed in PBS containing 0.02% Tween-20 (PBST) in preparation for IHC. Deparaffinization/Antigen Retrieval (FFPE tissues): slides were immersed in 1x Universal Decloaker Solution and heated to 90°C for 45 minutes in the decloaking chamber, then submerged in Hot Rinse 20 times, and equilibrated with distilled water. Autostainer: slides were treated with Peroxidazed and blocked with Background Sniper prior to incubating with the primary antibody in Da Vinci Green Diluent for 30 minutes. The anti-MMP9 (ab76003), anti-collagen IV (ab6586), and anti-PM2K (ab58822) antibodies were obtained from Abcam (Cambridge, MA). The anti-myeloperoxidase (MPO, A0398) was obtained from Dako (Carpinteria, CA). The slides were then rinsed in TBS-Autowash. The Mach 2 polymer kit was used for antigen detection by adding anti-rabbit secondary antibody (conjugated to horseradish peroxidase) for 30 minutes. DAB (3, 3' diaminobenzidine) chromagen was added to the slides for 1 minute followed by a single rinse in TBS-Autowash and a single rinse in distilled water. Slides were then counterstained with CAT hematoxylin, followed by manual dehydration with graded alcohol, then mounted with entellan mounting media. All slides were visualized using a Leica DFC500 light microscope.

### Animal welfare

All studies conducted with mice or rats were carried out in strict accordance with the recommendations in the Guide for the Care and Use of Laboratory Animals of the National Institutes of Health and were approved by the local IACUC overseeing each facility where studies were conducted: Biomodels Instituional Animal Care and Use Committee (IACUC approval number 09-1215-03); Aragen Committee for Animal Care and Use (IACUC approval number SA-003-A); Animal Care and Use Committee at AntiCancer (IACUC approval number 14–090).

### Graphing and statistical analysis

Data were analyzed and visualized using Prism software (GraphPad, v5.01). For clinical, histopathological, and immunohistochemistry assessments, the significance of regulation of treatment groups vs. the vehicle group was assessed as follows: The D'Agostino & Pearson omnibus normality test was used to determine whether data were normally distributed. Data that were normally distributed were evaluated by a one-way ANOVA with Dunnett’s Multiple Comparison post-test. Non-normally distributed data were evaluated by either a Mann Whitney test (for pairwise analysis) or by a Kruskal-Wallis test with the Dunn’s Multiple Comparison post-test. Fisher’s exact test was used for analysis of metastases data. P value designations are as follows: * < 0.05, ** < 0.01, *** <0.001, **** < 0.0001.

## Results

### Generation and characterization of anti-MMP9 antibodies

**Fig 1:**
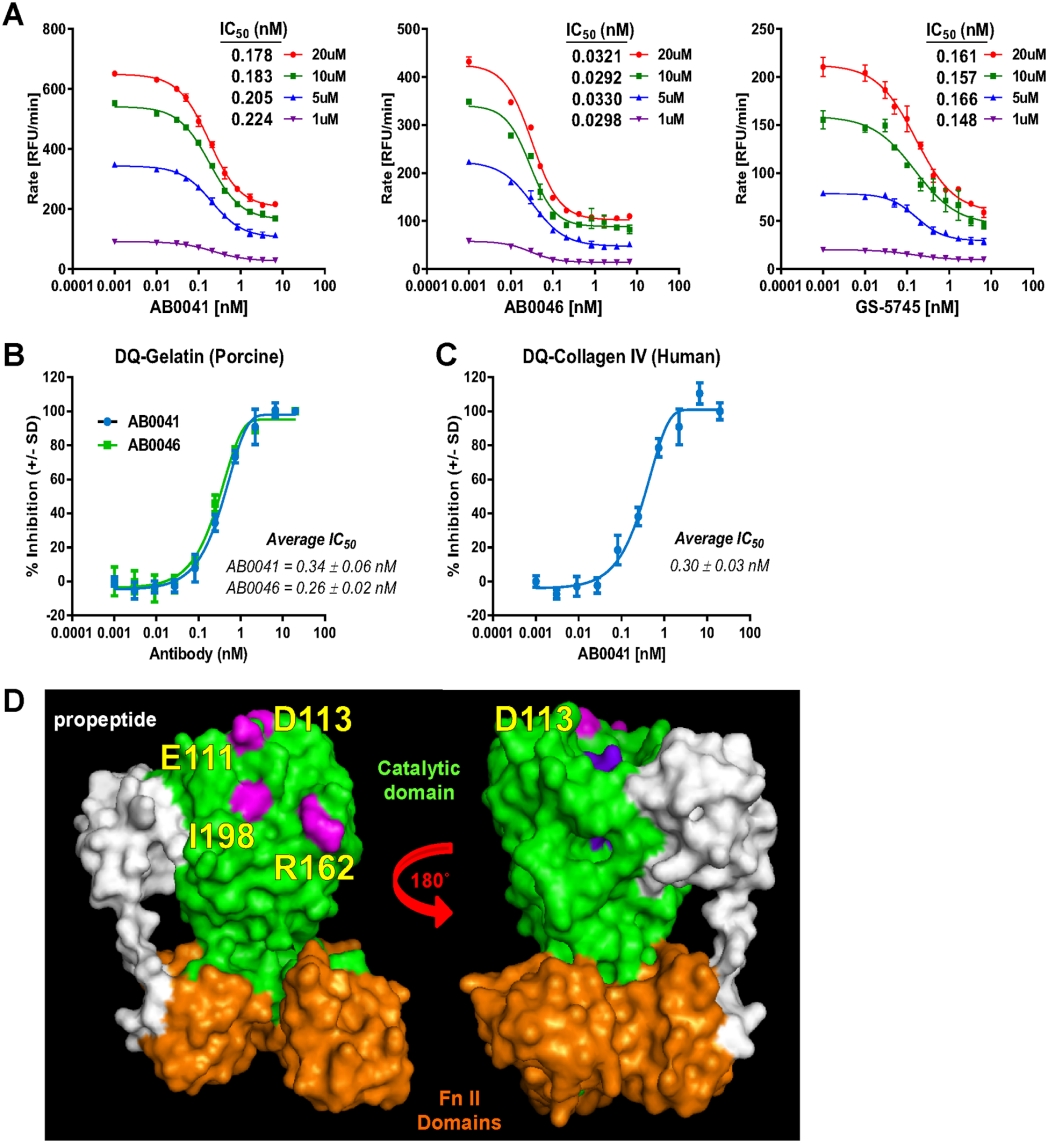
Characterization of the mode of inhibition and binding epitopes for anti-MMP9 antibodies. **(A)** AB0041, AB0046, and GS-5745 demonstrate noncompetitive inhibition of MMP9: Antibody-mediated inhibition of MMP9 activity was assessed at multiple concentrations of substrate (a fluorogenic peptide). **(B)** AB0041 inhibition of human DQ-gelatin was evaluated over a dilution series of antibody concentration. Shown are a representative curve and the average ± standard deviation of three independent experiments. **(C)** AB0041 inhibition of human DQ-collagen IV was evaluated over a dilution series of antibody concentration. Shown are a representative curve and the average ± standard deviation of three independent experiments. **(D)** Structural model of MMP9. Residues identified as involved in AB0041 binding to MMP9 are shown in pink (including the key residue R162) and are distinct from residues engaging the active site Zn^++^ (shown in purple).

MMP9-targeted monoclonal antibodies were generated by immunizing mice with recombinant human or mouse MMP9 proteins, and candidate antibodies were identified by *in vitro* screening for target binding, inhibition of substrate proteolysis, and selectivity vs. other MMP family members. The selection process yielded AB0041, which inhibits both human MMP9 (hMMP9) and rat MMP9 (rMMP9), but does not bind to mouse MMP9 (mMMP9); and AB0046, which conversely inhibits mMMP9, while not binding to rMMP9 or hMMP9 (Table 1). Both antibodies had high affinity and excellent potency: for AB0041 targeting hMMP9, K_D(app)_ = 0.133 ± 0.030 nM and IC_50_ = 0.172 ± 0.007 nM, and for AB0046 targeting mMMP9, K_D(app)_ = 0.218 ± 0.097 nM and IC_50_ = 0.029 ± 0.005 nM (Table 1). AB0041 and AB0046 were highly selective, with greater than 500-fold selectivity for MMP9 vs. other MMP family members (including the highly homologous MMP2) (Table 1 and Table 2). Both antibodies behaved as non-competitive inhibitors, as the IC_50_ values generated against the enzyme activity on a peptide substrate were not substantially affected by substrate concentrations ranging from 1–20 μM (Fig 1A). Additionally, both antibodies inhibited MMP9-mediated cleavage of the physiologically relevant substrates gelatin and basement membrane collagen IV, with potencies similar to those generated in the peptide substrate assay. The IC_50_ of AB0041 was 0.34 ± 0.061 nM for gelatin cleavage (Fig 1B) and 0.30 ± 0.025 nM for collagen IV cleavage (Fig 1C), and the IC_50_ of AB0046 for gelatin cleavage was 0.26 ± 0.018 nM (Fig 1B). Mouse MMP9 did not digest human collagen IV, so AB0046 was not assessed in this assay. The ability of AB0041 and AB0046 to inhibit MMP9-mediated cleavage of gelatin (partially denatured collagen) suggests that the antibodies could be effective in preventing liberation of extracellular matrix sequestered cytokines and growth factors, and the ability of AB0041 to inhibit collagen IV degradation is notable in that it provides direct evidence of the antibody’s potential to inhibit MMP9-mediated epithelial and endothelial basement membrane degradation.

**Table 1:** Characterization of the affinity, species specificity, and potency of anti-MMP9 antibodies.

	AB0041		AB0046		GS-5745	
Antigen	K_d(app)_ (nM) ^a^	IC_50_ (nM) ^a^	K_d(app)_ (nM) ^a^	IC_50_ (nM) ^a^	K_d(app)_ (nM) ^a^	IC_50_ (nM) ^a^
Human MMP9	0.133 ± 0.030	0.172 ± 0.007	>100	>100	0.168 ± 0.117	0.218 ± 0.040
Rat MMP9	0.332 ± 0.022	4.1 ± 2.03	>100	>100	0.311 ± 0.017	7.4 ± 1.24
Mouse MMP9	>100	>100	0.218 ± 0.097	0.029 ± 0.005	>100	>100

^a^. Data is presented in the format of X ± Y, where X is the mean value of and Y is the standard deviation of three independent experiments

**Table 2:** Characterization of the affinity, species specificity, and potency of anti-MMP9 antibodies.

MMP	AB0041 K_d(app)_ (nM)	AB0046 K_d(app)_ (nM)	GS-5745 K_d(app)_ (nM)
MMP1	>100	N.D.	>100
MMP2	>100	>100	>100
MMP3	>100	>100	>100
MMP7	>100	>100	>100
MMP8	>100	>100	>100
MMP10	>100	N.D.	>100
MMP12	>100	>100	>100
MMP13	>100	N.D.	>100
MMP14	>100	N.D.	>100
MMP16	>100	N.D.	>100

The inability of AB0041 to bind mMM9 or of AB0046 to bind hMMP9, despite a high degree of sequence identity (75%) between both species, afforded us a means to identify their binding epitopes. We first determined that the antibodies bound to a region in the pro-catalytic domains of hMMP9 or mMMP9, rather than to the hemopexin domains. We then used a published crystal structure for hMMP9 [90] to select surface-exposed amino acids that differed between human and mouse, individually mutated these residues to match the opposite species, and screened for the ability of each mutation to initiate binding by AB0041 or AB0046. The P162R mutation in mMMP9 resulted in the most prominent gain-of-binding by AB0041 (S1A Fig), suggesting that R162 in hMMP9 is a critical residue for AB0041 engagement. In addition, three other residues (E111, D113, I198) were identified during epitope mapping and are highlighted in the crystal structure of human MMP9 (Fig 1D, S1A and S1B Fig). These four residues are in the vicinity of the enzymatic domain Zn^2+^ ion, but do not surround the substrate binding (catalytic) pocket (Fig 1D), consistent with the antibody’s apparent non-competitive mode of inhibition. Interestingly, mutating R162 in hMMP9 to the analogous mouse residue (P) was sufficient to initiate binding to AB0046 (S1C Fig), suggesting that both antibodies recognized similar epitopes on MMP9.

### Evaluation of anti-MMP9 antibodies in a preclinical model of musculoskeletal syndrome

**Fig 2:**
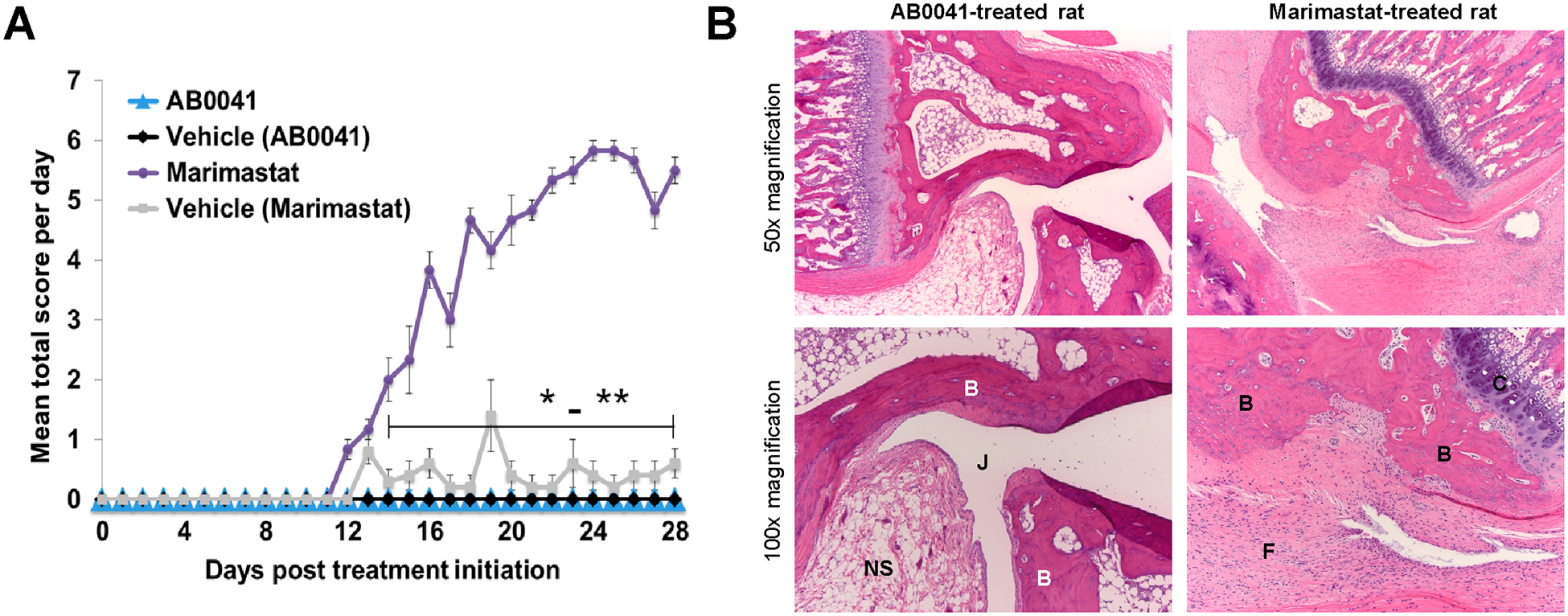
Effects of AB0041 on rat joints. (A) Assessment of AB0041 (50 mg/kg, twice daily) and marimastat (5.5–7 mg/kg/day) in an MSS model: Mean total disease scores (+/- SD) of rats treated with AB0041, marimastat, or vehicle are shown. Marimastat and AB0041 groups each have a matched vehicle group. (B) Representative 50x and 100x images of H&E-stained sections of joints from AB0041- or marimastat-treated rats demonstrate evidence of joint disease with marimastat treatment but not with AB0041 treatment. Significance was assessed with a Mann-Whitney test. P value designations are as follows: * < 0.05, ** < 0.01, *** <0.001, **** < 0.0001.

The association of certain non-selective MMP inhibitors, such as marimastat, with the development of musculoskeletal syndrome (MSS) [17] prompted us to assess the effects of selective MMP9 inhibition by AB0041 (which binds to and inhibits rat MMP9, Table 1) in a rat model of this disorder [88]. Treatment of rats with marimastat results in symptoms that parallel those of the human syndrome, including the development of synovial hyperplasia and increased cellularity in joints, as well as compromised ability to rest on hind feet, inability to move, and high-stepping gait [88]. In our study, the first signs of musculoskeletal disease were evident in marimastat-treated rats 12 days after the initiation of dosing, and these symptoms rapidly worsened from days 12 to 18. Disease severity was assessed by a clinical total score that was composed of the sum of a gait score, resting posture score, and willingness-to-move score for each animal. The mean total score of the marimastat-treated group was significantly elevated vs. that of the matched vehicle-treated group from day 14 onwards (Fig 2A). In contrast, rats treated with AB0041 did not show any symptoms of musculoskeletal disease during the course of the study (Fig 2A). Histopathologic analysis of joints was consistent with the clinical observations in that it revealed moderate to severe inflammation and fibrosis in the marimastat-treated group, but no sign of disease in the AB0041-treated group (Fig 1B and S2A Fig). Serum titer analysis of AB0041 levels throughout the study confirmed sustained exposure to the antibody at an average concentration of greater than 2 mg/ml (13 μM) (S2B Fig), which is well above the *in vitro* IC_50_ for inhibition of rMMP9 activity (Fig 1A). We confirmed the ability of AB0041 to enter the joints and target rMMP9 by evaluating it in a preliminary trial of a rat collagen-induced arthritis (CIA) model, which exhibits MMP9 expression in macrophages and osteoclasts in the diseased joints (S2C Fig). AB0041 treatment (50 mg/kg twice weekly) for 14 days after establishment of disease significantly reduced the cumulative pathology sum score of joints to non-diseased control levels (S2D Fig).

### MMP9 expression and association with disease in human UC and in mouse DSS colitis

**Fig 3:**
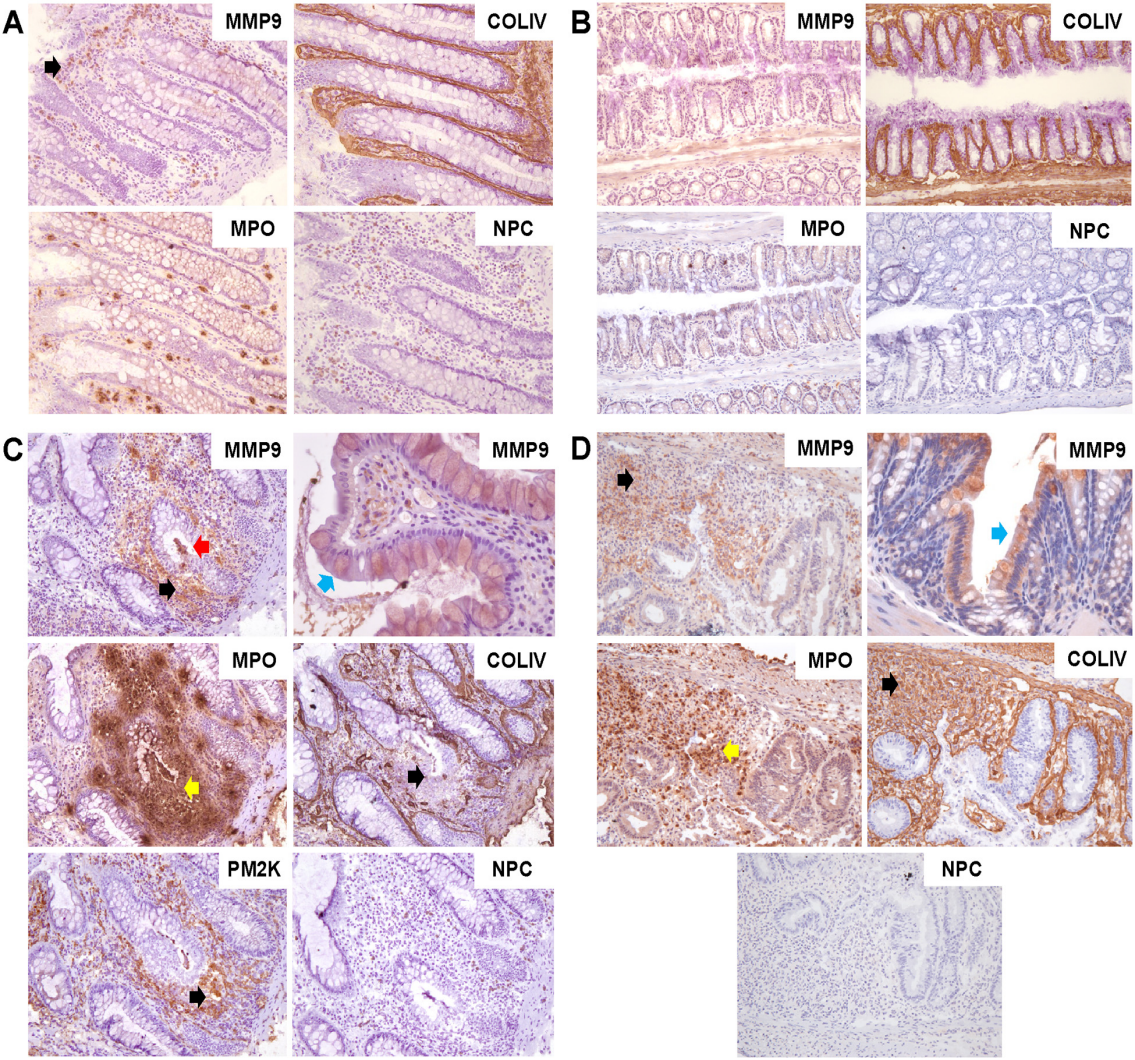
MMP9 expression and association with disease in ulcerative colitis and in DSS-induced colitis. IHC was conducted on serial sections of frozen human non-diseased colon tissue (from healthy individuals, or in non-diseased colonic crypts found adjacent to diseased regions in UC patient samples, n = 12 samples; representative images shown) (A) or of mouse non-diseased colon tissue (n = 2) (B). MMP9 immunoreactivity was limited, and consisted primarily of cytoplasmic staining of a subset of immune cells such as macrophages/histiocytes, lymphocytes, and neutrophils within the lamina propria and submucosal regions of the colon (black arrow). (C) IHC was conducted on serial sections of frozen human UC patient colon tissue (n = 7 patients; representative images shown) and demonstrated MMP9 induction at a disease focus (top left image, black arrow) surrounding an abscessed epithelial crypt (red arrow), and MMP9 expression coincident with regions of neutrophil (MPO, middle left image, yellow arrow) and macrophage (PM2K, bottom left image, black arrow) infiltration/expansion, as well as with regions of disrupted epithelial basement membrane (COLIV, bottom right image, black arrow). MMP9 induction in colonic epithelium was also observed (top right image, blue arrow, 400x). (D) IHC conducted on serial sections of frozen mouse DSS colitis tissue (n = 9) showed an MMP9 expression pattern similar to human UC, with MMP9 induction in a region of inflammation surrounding diseased epithelial crypts (top left image, black arrow), MMP9 induction in colonic epithelium (top right image, blue arrow, 400x), neutrophil infiltration at the active disease site (MPO, bottom left image, yellow arrow) and disruption of the epithelial basement membrane in a disease area (COLIV, bottom right image, black arrow). All images are at 200X magnification unless otherwise noted. NPC = no primary antibody control.

Having determined the potency and selectivity of our MMP9-targeting preclinical antibodies, we next sought to evaluate the therapeutic potential of MMP9 inhibition in ulcerative colitis, an autoimmune disease that is characterized by elevated circulating levels of MMP9 and of MMP9 expression locally, at sites of active disease [11, 12]. We chose the dextran sodium sulfate (DSS)-induced colitis model [91], which has pathological features similar to that of human UC, and can mimic the human disease progression (i.e. inflammation-dysplasia-adenocarcinoma) upon chronic DSS administration [92, 93]. Mice that ingest DSS develop inflammation of the colonic mucosa and exhibit colonic crypt destruction that, as in human UC, results in bloody diarrhea and weight loss. Immunohistochemical (IHC) analysis of colon tissue from healthy human donors (Fig 3A) and from healthy mice (Fig 3B) showed that MMP9 expression consisted primarily of cytoplasmic staining and was confined to a subset of macrophages, neutrophils, and lymphocytes within the lamina propria and submucosal regions. In contrast, strong MMP9 expression was evident at disease foci in UC (Fig 3C) and in DSS-colitis tissue (Fig 3D), including in abscessed and necrotic crypts and regions of cryptitis, as well as in the lamina propria. MMP9 expression was prominent in neutrophilic infiltrates (MPO, Fig 3C and 3D) which are greatly induced in diseased regions versus healthy colon (Fig 3A and 3B), and was also identified in a subset of macrophages (PM2K, Fig 3C). Extracellular MMP9 immunoreactivity co-localized with regions of destruction of epithelial and endothelial basement membrane collagen IV (COLIV, Fig 3C and 3D) and intracellular MMP9 expression was evident in mucosal epithelial cells in diseased regions (Fig 3C and 3D, upper right panel), consistent with previous reports [11, 74].

### Efficacy of therapeutic dosing of AB0046 in a mouse DSS colitis model of UC

**Fig 4:**
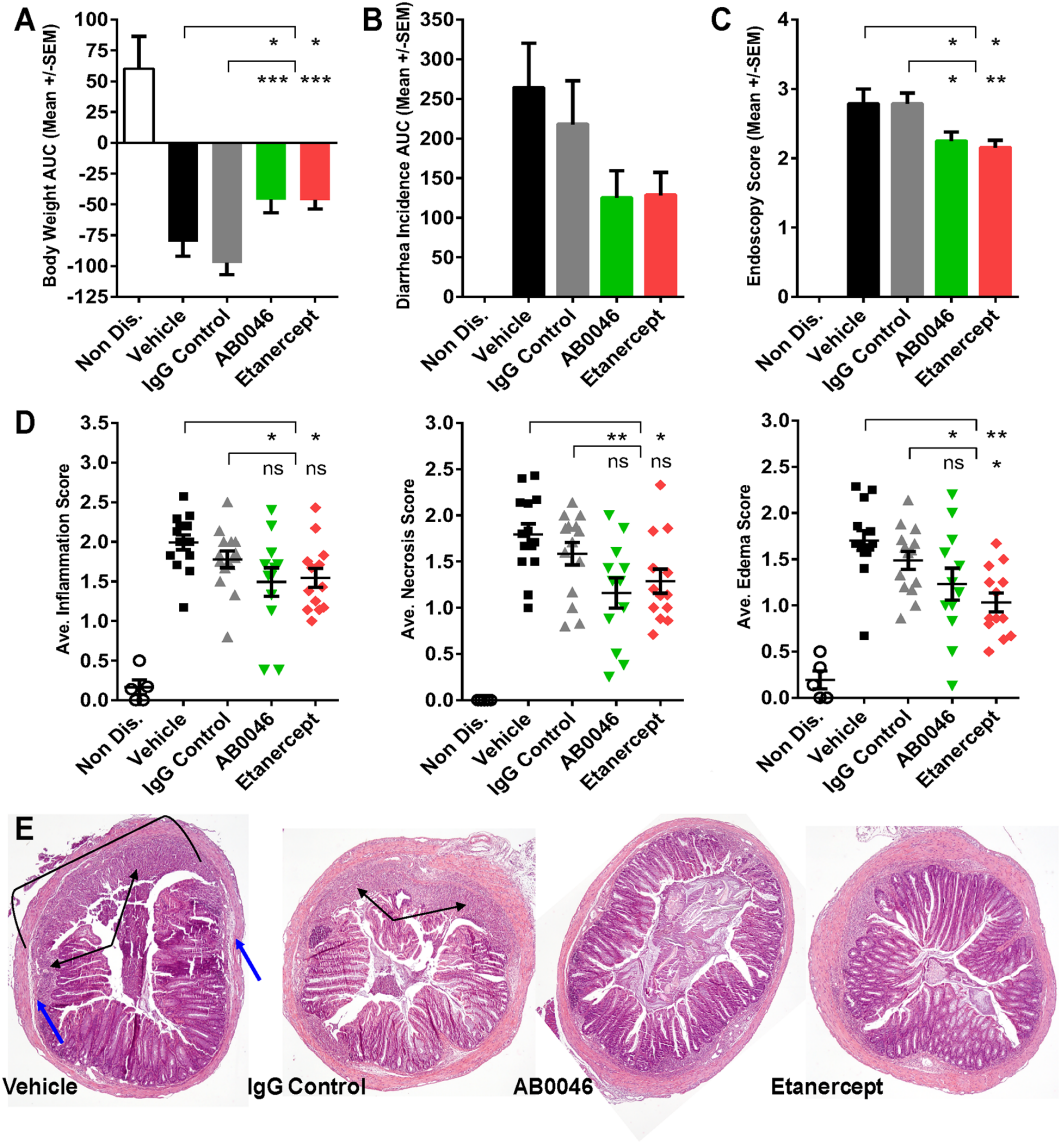
Efficacy of MMP9-targeting antibody in mouse DSS model of colitis. Treatment was initiated after establishment of colitis (Day 6): Vehicle control, IgG control (30 mg/kg), and AB0046 (30 mg/kg) were dosed every three days, and entanercept (10 mg/kg) was dosed every two days (A) The area under the curve (AUC) was calculated for daily body weight changes in each animal by the trapezoidal rule method;$Area=\left(t_{2}-t_{1}\right)\left[\frac{\int \left(t_{1}\right)+\int \left(t_{2}\right)}{2}\right]$. (B) The incidence of diarrhea was recorded daily and the AUC calculation was performed as above. (C) Endoscopic evaluation was performed on all groups at study termination. Scoring was based on the single most severe lesion observed in the distal 5 cm of colon. (D) Blinded histopathological analysis was performed on colons excised at study termination. The degree of inflammation (primarily macrophages and neutrophils), edema, and necrosis was scored. (E) Images representative of study groups (40X magnification) were taken by a pathologist and highlight areas of inflammation/mucosal necrosis (black arrows) and edema (blue arrows), which are reduced in AB0046 and etanercept-treated animals. Statistical significance was assessed by one-way ANOVA with Dunnett’s Multiple Comparison post-test. P value designations are as follows: * < 0.05, ** < 0.01, *** <0.001, **** < 0.0001.

We assessed the effect of MMP9 inhibition on established colitis by starting treatment with the mouse-specific antibody AB0046 after a 5-day course of DSS administration via the drinking water. Etanercept, an anti-TNF agent that is cross-reactive with mouse TNF-α [94], was used a reference compound in the study. The results of AB0046 treatment on clinical measures of disease were similar to those of etanercept: significant protection against body weight loss (Fig 4A), a reduction in the incidence of diarrhea by approximately 40% (Fig 4B), and a significant improvement in mean endoscopy scores (Fig 4C) when compared with isotype control. AB0046 treatment also decreased histological disease as measured by reduction in tissue inflammation, mucosal necrosis, and edema scores to levels comparable to etanercept treatment (Fig 4D and 4E). Histological changes reached significance vs. vehicle but not vs. isotype control antibody. We are currently investigating whether similar anti-inflammatory effects are seen with alternate isotype control antibodies in this model. In addition, both AB0046 and entanercept treatment resulted in a clear preservation of epithelial crypt architecture (Fig 4E). Interestingly, ELISA analysis of colon tissue lysates revealed a 55% reduction of total MMP9 levels in the AB0046-treated group vs. the vehicle group, while MMP9 levels in the etanercept group were similar to vehicle control (S3A Fig). Prophylactic dosing of AB0046 in the mouse DSS-induced colitis model, starting one day prior to DSS administration, also reduced diarrhea incidence and significantly improved histopathological disease (S3B and S3C Fig).

### Anti-MMP9 antibody reduces soluble TNF-α generation

**Fig 5:**
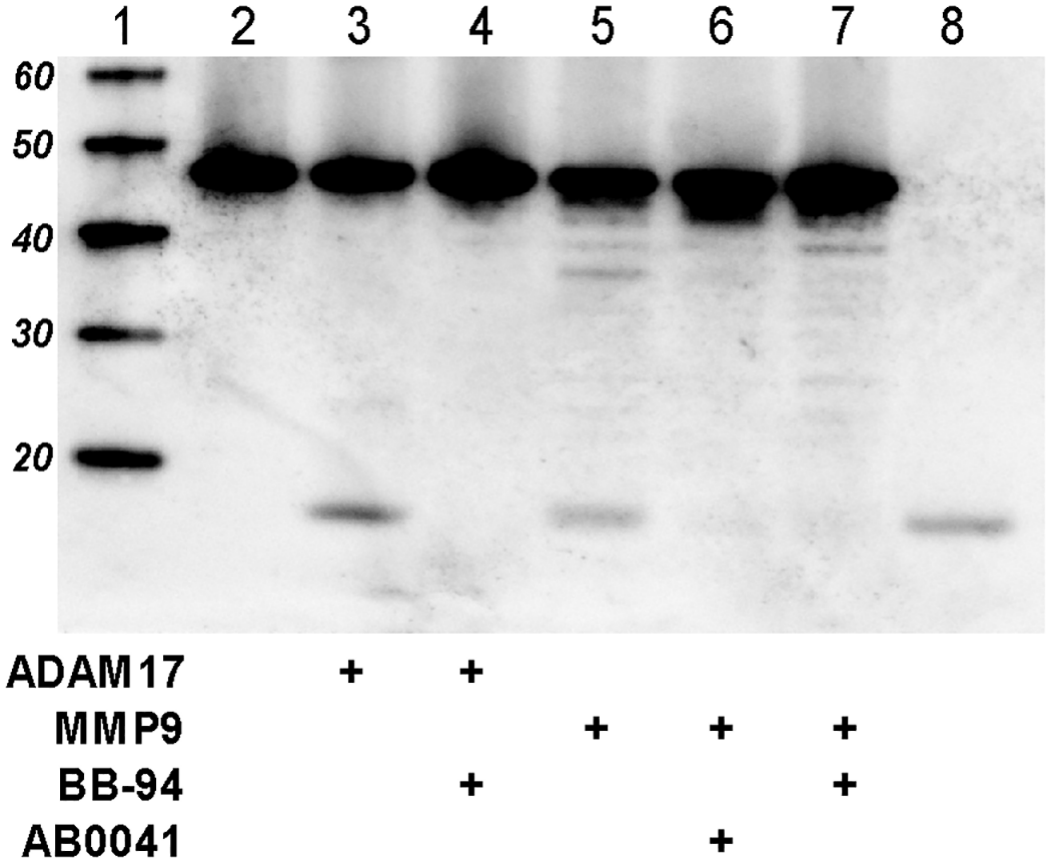
Anti-MMP9 antibody reduces soluble TNF-α generation. To assess cleavage, 2 μg of pro-TNF-α was incubated with either 2 μg ADAM17 or 2 μg MMP9 in the presence or absence of inhibitor. Cleavage reactions were allowed to incubate overnight at 37°C and were then analyzed by immunoblotting. Lanes are as follows: 1. Molecular weight markers, 2. Pro-TNF-α alone, 3. Pro-TNF-α + ADAM17, 4. pro-TNF-α + ADAM17 + BB-94 (10 μM), 5. Pro-TNF-α + MMP9, 6. Pro-TNF-α + MMP9 + AB0041 (10 μM), 7. Pro-TNF-α + MMP9 + BB-94 (10 μM), 8. Soluble TNF-α.

We next evaluated whether anti-MMP9 antibody was able to inhibit release of soluble TNF-α (sTNF-α) and thereby, potentially elicit local anti-inflammatory effects. A recombinant TNF-α fusion protein was cleaved by a soluble version of ADAM17 (a well-characterized TNF-α converting enzyme, [95]) as well as by APMA-activated MMP9, to generate the 17kDa sTNF-α. The non-selective metalloproteinase antagonist BB-94 inhibited release of sTNF-α by ADAM17, and both BB-94 and AB0041 inhibited release of sTNF-α by MMP9 (Fig 5).

### MMP9 expression in human colorectal carcinoma (CRC) and in a preclinical mouse model of CRC

**Fig 6:**
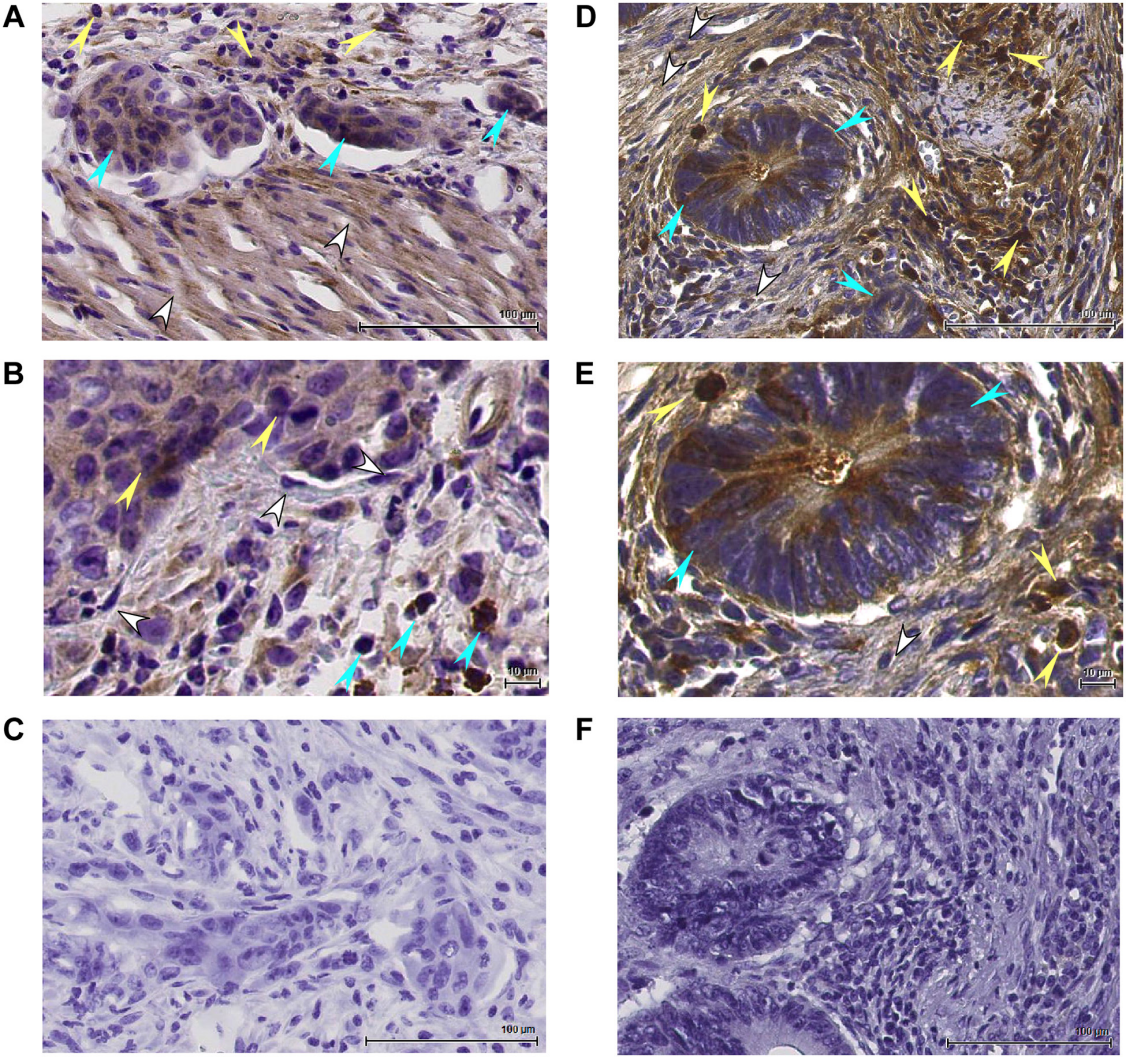
MMP9 expression in human CRC and in an orthotopic xenograft mouse model of CRC. IHC analysis of HCT116-derived xenograft tumors (A, B) or of human CRC tumors (D, E). MMP9 staining from various cellular sources is highlighted as follows: blue arrows, tumor cells; yellow arrows, inflammatory cells; white arrows, stromal cells such as fibroblasts or smooth muscle cells. (A, B) Immunohistochemical staining for MMP9 in HCT116-derived tumors at 200x (A) or 400x (B) magnification. (D, E) Immunohistochemical staining for MMP9 in a human colorectal carcinoma at 200x (D) or 400x (E) magnification. Panels C (HCT116-derived tumors) and F (human CRC) show tissue sections that were incubated with secondary antibody only and demonstrate the absence of non-specific secondary antibody binding (200x magnification).

Since chronic UC is a risk factor for CRC, and since MMP9 expression is associated with poor prognosis in CRC patients [35–38], we were also interesting in assessing the therapeutic benefit of MMP9 inhibition in a model of CRC. We chose a surgical orthotopic HCT116 xenograft model (rather than a subcutaneous/flank xenograft model) in order to better recapitulate key histological features of CRC. IHC analysis of tumors from vehicle-treated mice in the HCT116 model (Fig 6A and 6B) demonstrated that MMP9 expression in the xenograft tumor was generally analogous to that of human CRC (Fig 6D and 6E): In both cases, MMP9 was localized extracellularly in regions of desmoplasia and was produced by a subset of tumor cells, by non-inflammatory stromal cells such as fibroblasts and endothelial cells, and by inflammatory stromal cells such as macrophages and/or neutrophils.

### Efficacy of anti-MMP9 antibodies in a preclinical model of CRC

**Fig 7:**
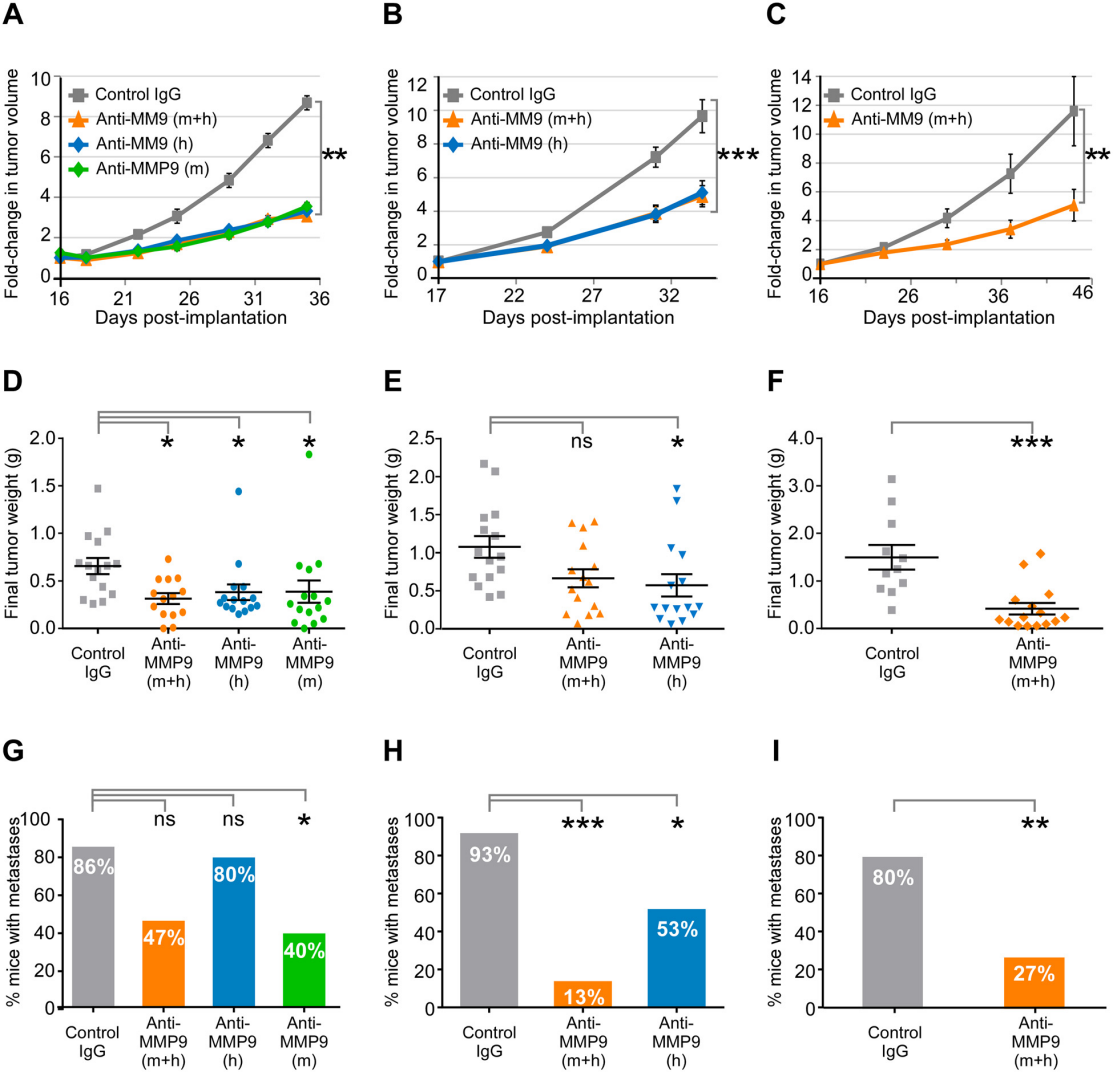
Efficacy of MMP9 inhibition in an orthotopic xenograft model of CRC. Tumors in the HCT116 model were allowed to reach a volume of 70–100 mm^3^ before treatment with a control IgG or with antibodies targeting mouse MMP9 (AB0046; anti-MMP9 [m]), human MMP9 (AB0041; anti-MMP9 [h]), or both (anti-MMP9 [m+h]). Each antibody was dosed twice a week at 15 mg/kg, whether singly or in combination, and study groups containing AB0046 received a loading dose of 50 mg/kg on the day prior to initiation of treatment. Three separate studies are shown; Study 3 (panels A, D, G); Study 2 (panels B, E, H), and Study 1 (panels C, F, I). (A-C) Change in tumor growth: (A) Study 3, (B) Study 2, (C) Study 1. For a given mouse, raw tumor volume measurements (by caliper) were normalized to the corresponding initial tumor volume (prior to the start of treatment initiation). Normalized volumes for individual mice were then averaged for each timepoint;plots show group mean +/- SEM; standard error of the mean. Significance was assessed by Kruskal-Wallis analysis (A, B) or by Mann-Whitney analysis (C). Treatment was initiated 16 days after surgical implantation of tumor fragments for Studies 1 and 3, and 17 days after implantation for Study 2. The last measurement of tumor volume was 35 days post-implantation for Study 3, 34 days post-implantation for Study 2, and 44 days post-implantation for Study 1. (D-F) Final tumor weight: (D) Study 3, (E) Study 2, (F) Study 1. Plots show mean tumor weight +/- SEM. Significance was assessed by Kruskal-Wallis analysis (D,E) or by Mann-Whitney analysis (F). Mice were terminated at 20 days (Study 3), 18 days (Study 2), or 32 days (Study 1) after treatment initiation, which corresponds to 36 days (Study 3), 35 days (Study 2), or 48 days (Study 1) after tumor implantation. (G-I) Studies 1–3; metastases incidence at study termination: (G) Study 3, (H) Study 2, (I) Study 1. Metastases were scored as present or absent, based on open-imaging visualization of the GFP-labeled HCT116 tumor cells at areas distal to the primary tumor mass. Plots show the percentage of mice displaying metastases per group. Significance was assessed by Fisher’s exact test. For all panels in Fig 7, P value designations are as follows: * < 0.05, ** < 0.01, *** <0.001, **** < 0.0001.

The differing species-specificity profiles of our preclinical antibodies provided us with a unique opportunity to compare the effects of inhibiting tumor-derived (human) and stromal-derived (mouse) MMP9 either singly or in combination in the HCT116 xenograft tumor model. When surgically implanted tumors reached a volume of ~70–100 mm^3^, mice were injected with AB0041 (which targets human MMP9), with AB0046 (which targets mouse MMP9), with a 1:1 mixture of AB0041 and AB0046, or with an isotype control antibody. We performed three successive studies using this model: Study 1 (Fig 7C, 7F and 7I), Study 2 (Fig 7B, 7E and 7H), and Study 3 (Fig 7A, 7D and 7G). We initially tested dual inhibition of hMMP9 and mMMP9 via combined treatment with AB0041 + AB0046 (Study 1; anti-MMP9 [m+h]); in subsequent studies we also tested single-agent AB0041 (Study 2; anti-MMP9 [h]) and single-agent AB0046 (Study 3; anti-MMP9 [m]). Interestingly, inhibition of hMMP9 or mMMP9 either singly or in combination yielded significant reductions in tumor growth rate (Fig 7A, 7B and 7C) and in final tumor weight (Fig 7D, 7E and 7F) vs. the isotype-control group (inhibition with anti-MMP9 [m+h] approached, but did not achieve, significant tumor weight reduction in Study 2/Fig 7E). These data suggest that both stromal-derived and tumor-derived MMP9 contribute to primary tumor growth, although surprisingly, we did not observe additive or synergistic efficacy in the dual-antibody group vs. the single-antibody groups. This may be due to the already substantial efficacy of each single agent (i.e. little to no window for further reduction in this model), and/or to the presence of residual necrotic tumor tissue, which might have prevented further reduction in tumor size. Inhibition of MMP9 also limited the ability of the primary tumor to colonize distal sites: mice in the anti-MMP9 (m+h) treatment group in Studies 1 and 2 (Fig 7I and 7H) had significantly reduced metastasis vs. the isotype control—and in Study 3 (Fig 7G), the metastases reduction in the anti-MMP9 (m+h) group closely approached significance (p = 0.0502 vs. isotype control). It’s notable that inhibition of tumor-derived (human) MMP9 alone was not as effective in metastases reduction as dual targeting of both tumor and stromal MMP9 (see Fig 7G and 7H), while inhibition of stroma-derived MMP9 (mouse) was as effective as dual targeting (see Fig 7G). The observed difference in anti-metastases efficacy is consistent with evidence supporting a prominent role for stromal MMP9 in metastatic growth [65, 70, 73, 96, 97].

### Humanization of AB0041

Given the promising therapeutic potential demonstrated in the UC and CRC preclinical models, we generated a humanized antibody for use in human clinical trials. The variable domains from the IgG and kappa chains of the murine antibody AB0041 were humanized via a proprietary de-immunization strategy and were cloned into a human IgG4 heavy chain with S241P hinge-stabilizing mutation [98, 99] and a human kappa light chain, respectively (Antitope Ltd, Cambridge, UK), to generate the clinical candidate GS-5745. When evaluated using *in vitro* assays, GS-5745 showed potency and selectivity equivalent to that of AB0041 (Table 1 and Table 2), and also exhibited a non-competitive mode of inhibition of MMP9 (Fig 1A), confirming that these key therapeutic properties were preserved during humanization.

## Discussion

The pathologies associated with dysregulation of MMP9 expression in both human diseases and animal models of disease support the long-standing interest in MMP9 as a therapeutic target [100–102]. In this report, we describe development of a highly selective allosteric anti-MMP9 antibody that demonstrates a noncompetitive mode of inhibition, which we believe is a therapeutic advantage, given the high levels of MMP9 substrates *in vivo* [103]. To our knowledge, only one other MMP9 inhibitor that can discriminate between MMP9 and MMP2 has been reported (the REGA-3G12 antibody) [104].

The prevalence of upregulated MMP9 in inflammatory indications such as UC suggests that a selective inhibitor of MMP9 could have substantial therapeutic impact: UC patients represent an unmet clinical need, since none of the current therapies result in widespread remission or in mucosal healing [44, 105] and because UC patients are at an increased risk for CRC [15, 31–33]. Anti-TNF therapeutics target an important driver of the chronic inflammatory response [106, 107] and have had success in the clinic, but patients on this therapy are at increased risk of infection [108], and 80% fail to achieve long-term remission and relapse within one year [109]. We suggest that a highly selective inhibitor of MMP9 has advantages over current treatment options for UC patients, because targeting this disease-associated downstream mediator of inflammation and tissue destruction may have superior safety and a larger therapeutic index than immunosuppressive agents.

MMP9 is implicated as a primary disease driver in UC because of its abundant disease-specific expression. Our IHC analysis of human UC tissue showed that both macrophages and neutrophils, which are characteristic of the inflammatory infiltrate in this disease [14, 16], are closely associated with MMP9 expression in intestinal lesions. Since neutrophil degranulation releases MMP9 without the typical co-secretion of endogenous inhibitor TIMP1 [110], these cells can deliver a large amount of cleavage-competent enzyme directly at the disease site. Our animal model and *in vitro* data suggest that inhibition of disease-induced MMP9 can protect against generation of a local pro-inflammatory environment and tissue destruction, and could thereby be effective at creating conditions compatible with mucosal healing.

While rodent DSS-induced colitis does not model the autoimmune etiology of UC [13], it is a robust model for the role of MMP9 in colitis. The pathology and symptoms of UC and DSS colitis are strikingly similar—as is the expression pattern for MMP9, which positions MMP9 to act similarly in both cases to perpetuate and exacerbate disease subsequent to the initiating insults (e.g. via release and activation of growth factors/cytokines and basement membrane destruction). Therapeutic dosing of AB0046 in a DSS-induced colitis model of UC showed that inhibiting MMP9 after disease was established significantly improved multiple disease parameters, including histopathology and the clinically relevant metrics of body weight loss, diarrhea, and endoscopic disease. Inhibiting MMP9 during the establishment of disease by prophylactic dosing also showed benefit and no adverse effects, which is an important observation given the waxing and waning nature of human UC. While we saw a comparable degree of efficacy with AB0046 as with the anti-TNF agent etanercept, we measured a reduction in terminal colon tissue MMP9 levels (a measure of disease severity [11, 111]) with AB0046 but not with etanercept. Since AB0046 is a mouse IgG1 isotype, it has low effector activity and likely does not appreciably activate ADCC or other antibody-mediated clearance mechanisms. Therefore, we believe that the observed reduction in MMP9 levels is reflective of the inhibition of MMP9-mediated signaling cascades, resulting in less inflammatory activity and reduced MMP9 expression. In addition, we show data suggesting that one of the potential mechanism by which anti-MMP9 therapy may be eliciting anti-inflammatory effects is through reduced release of sTNF-α and reduction of TNF-α-driven signaling cascades.

Recently, Sela-Passwell et al. reported complementary data using a function-blocking antibody (SDS3) targeting both MMP9 and MMP2 in a DSS colitis model [58]. These authors reported efficacy in the DSS model similar to the efficacy we observed with AB0046; however, the SDS3 antibody was engineered to bind directly to the active site of MMPs (competitive inhibition), and was also reported to have cross-reactivity with MMP14. MMP2 inhibition in the context of colitis may have detrimental consequences, as MMP2 knockout mice demonstrate exacerbated DSS-induced colitis [76]. This suggests a protective role for MMP2 in the colon, and supports the idea that selectively targeting MMP9 alone is a safer therapeutic strategy.

A large body of data also supports targeting of MMP9 in CRC [35–38] and in other oncology indications [5, 6]: MMP9 has been investigated as a key player in metastasis and invasion for over two decades (e.g. see [112, 113]), and has subsequently been shown to be involved in other facets of cancerous growth, including priming of the metastastic niche and modulation of growth signaling [2, 5, 53, 64–68]. The important role of MMP9 in the growth and spread of tumors has also been highlighted by knockdown studies in murine models. Tumors generated using MMP9 shRNA-treated cancer cells were smaller and/or less metastatic than tumors generated with control cells [54, 114], and injection of MMP9-targeted siRNA into established tumors decreased tumor growth [115, 116]. In this report, we demonstrated that antibody-mediated inhibition of MMP9 reduced primary tumor growth and metastatic lesions in an orthotopic xenograft model of CRC, and we were also able to interrogate the efficacy of targeting tumor-derived and stroma-derived MMP9 either singly or in combination. We observed that both approaches led to a reduction in primary tumor growth, and we suggest that this intriguing functional reciprocity likely reflects the complex interplay of tumor-stroma signaling: tumor cells and tumor-associated macrophages in the stroma participate in a paracrine activation loop that stimulates the release of MMPs, cytokines, and chemokines, and all of these factors cooperatively promote and perpetuate a pro-tumor microenvironment [48–52]. However, the roles of tumor and stromal MMP9 in this model were not wholly interchangeable; inhibition of stromal MMP9 (whether alone or in combination with tumor-derived MMP9) was necessary to achieve maximal reduction of metastatic burden. These data highlight the potential division of labor between tumor-derived and stroma-derived MMP9 as well as the dependency between these two compartments in the process of tumorigenesis, and suggest that inhibition of MMP9-mediated proteolysis could be an effective means of dampening the tumor-stroma crosstalk that contributes to an oncogenic environment.

Collectively, these data support the therapeutic promise of an anti-MMP9 antibody in ulcerative colitis and colorectal cancer. We propose that selective MMP9 inhibition provides a unique opportunity to combat the tissue destruction, inflammation, cell migration, and mitogenic signaling that drive chronic inflammation and tumorigenesis. Phase I clinical trials for GS-5745 in UC and in solid tumors are currently ongoing (ClinicalTrials.gov identifiers NCT01831427, NCT02077465, and NCT01803282).

## Supporting Information

S1 ARRIVE Guidelines Checklist The ARRIVE guidelines checklist for reporting *in vivo* experimental data. (PDF)

S1 Fig Characterization of the selectivity and of the binding epitope for anti-MMP9 antibodies. **(A)** Gain-of-binding function of AB0041 was assessed by expressing point mutated versions of mMMP9 and evaluating by ELISA. **(B)** The table shows the mutations in mMMP9 that resulted in binding by AB0041, including the key mutation P162R. **(C)** AB0046 gain-of-binding to hMMP9 with the R162P mutation (ELISA analysis).
(PDF)

S2 Fig Effects of AB0041 on rat joints. **(A)** Summary of histopathology analysis of limbs from the MSS study, showing no disease in AB0041-treated animals and mild to moderate synovitis and fibrosis in marimastat-treated animals. **(B)** Serum AB0041 titers during the course of the MSS study, demonstrate sustained exposure of > 2 mg/ml. **(C)** H&E-stained sections, and IHC for CD68 (macrophage and osteoclast marker) and MMP9 in rat CIA hind limbs. Images from serial sections at 100x (left) and 400x (right) magnification show a diseased joint with MMP9 expression in macrophages in pannus tissue (white arrows) and osteoclasts on the surface of the eroding bone (red arrows). **(D)** Histopathology analysis of rat CIA hind limbs. AB0041 treatment (50 mg/kg, twice weekly) reduced limb pathology to levels similar to those of healthy controls, and showed equivalent efficacy to the reference agent methotrexate (MTX).
(PDF)

S3 Fig Efficacy of MMP9-targeting antibody in mouse DSS model of colitis. **(A)** MMP9 levels in mouse colon tissue were measured by ELISA. **(B)** The incidence of diarrhea was recorded and the AUC calculation was performed. **(C)** Blinded histopathological analysis was performed on colons excised at study termination. The degree of inflammation (primarily macrophages and neutrophils), of edema, and of necrosis was scored and a total pathology sum score was calculated. Statistical significance was assessed by one-way ANOVA with Dunnett’s Multiple Comparison post test (therapeutic study histopathology) or a Mann-Whitney test (prophylactic study histopathology). P value designations are as follows: * < 0.05, ** < 0.01, *** <0.001, **** < 0.0001.
(PDF)

S1 Methods Supplementary information materials and methods summary. This section includes information supporting materials and methods for the following: histopathology and titer analyses for the rat MSS model; study design, histopathology, and IHC for the rat CIA study; ELISA analysis and prophylactic study design for the mouse DSS-induced colitis model.
(PDF)
