# Supplementary material for: Selective Allosteric Inhibition of MMP9 Is Efficacious in Preclinical Models of Ulcerative Colitis and Colorectal Cancer
Source: PLoS One. 2015 May 11;10(5):e0127063. doi: 10.1371/journal.pone.0127063 (PMC4427291; doi:10.1371/journal.pone.0127063)

A

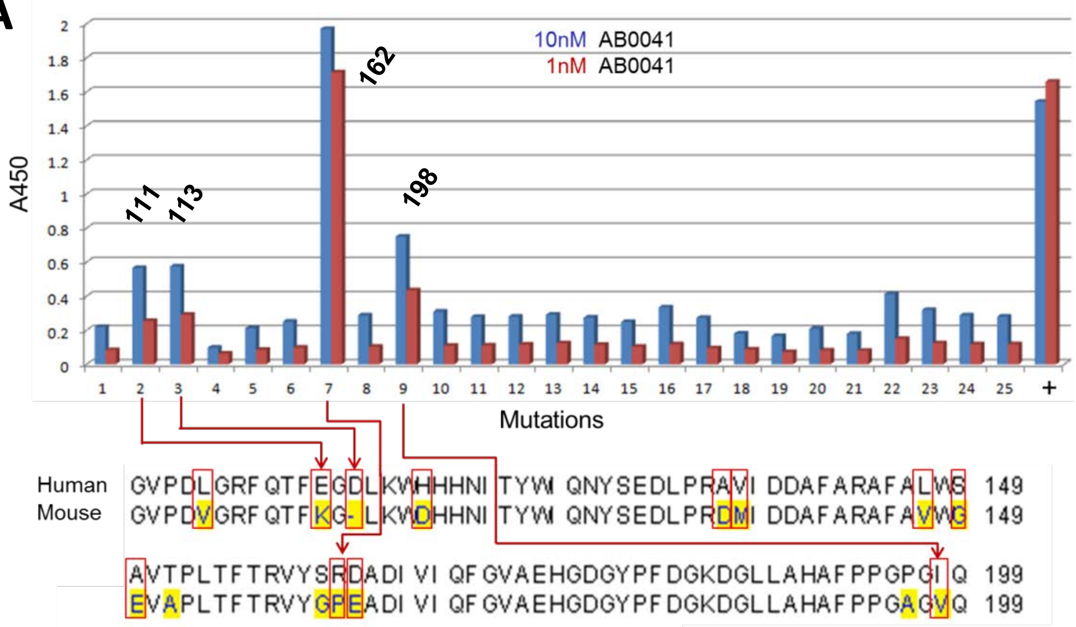

B

Mouse MMP9 Mutation	Analogous Human MMP9 Residue
K112E	E111
D insertion btn. G113 & L114	D113
P162R	R162
V198I	I198

C

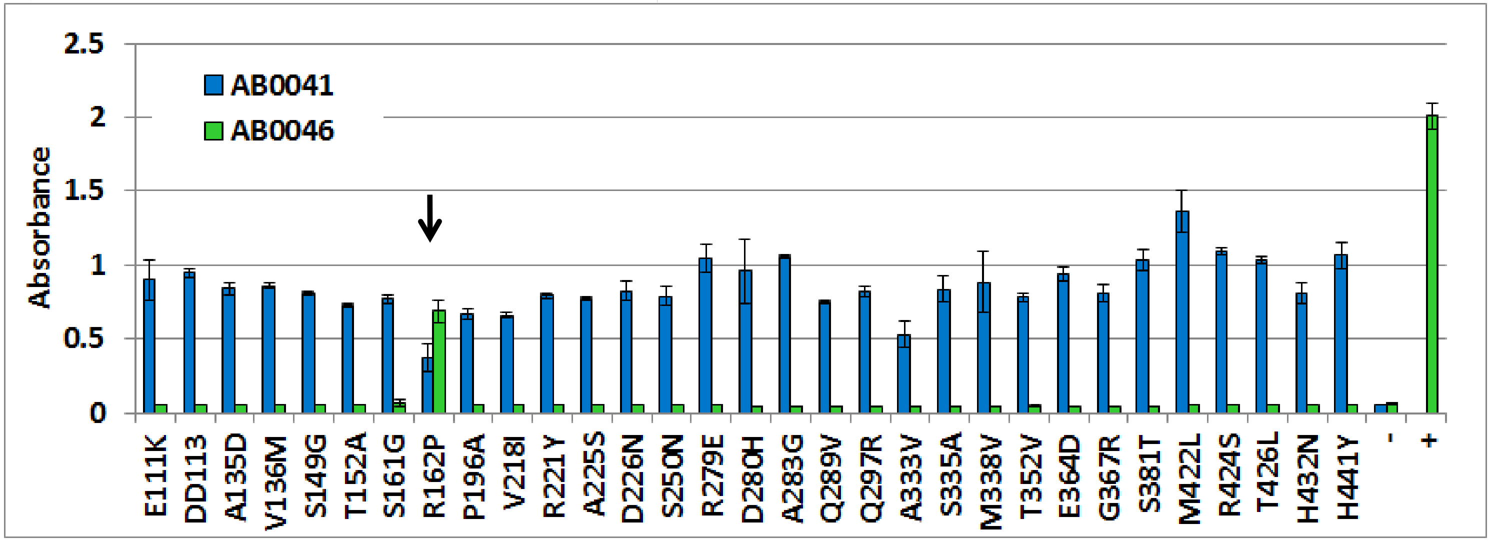

Supplement: S1 Fig — (A) Gain-of-binding function of AB0041 was assessed by expressing point mutated versions of mMMP9 and evaluating by ELISA. (B) The table shows the mutations in mMMP9 that resulted in binding by AB0041, including the key mutation P162R. (C) AB0046 gain-of-binding to hMMP9 with the R162P mutation (ELISA analysis). (PDF) [file pone.0127063.s002.pdf]
