# Supplementary material for: Selective Allosteric Inhibition of MMP9 Is Efficacious in Preclinical Models of Ulcerative Colitis and Colorectal Cancer
Source: PLoS One. 2015 May 11;10(5):e0127063. doi: 10.1371/journal.pone.0127063 (PMC4427291; doi:10.1371/journal.pone.0127063)

A

Summary of Changes in Limbs

Groups	AB0041	Marimastat[a]
Number of Animals	6	6
**Right Hind Knee**		
Synovitis	0	6 (1.8)
Fibrosis	0	3 (2.0)
Cartilage	0	1 (2.0)
**Right Hind Ankle**		
Synovitis	0	6 (1.5)
Fibrosis	0	6 (3.0)
**Left Hind Knee**		
Synovitis	0	4 (2.0)
Fibrosis	0	2 (2.0)
**Left Hind Ankle**		
Synovitis	0	4 (1.8)
Fibrosis	0	6 (3.0)
**Right Forelimb Elbow**		
Synovitis	0	0
Fibrosis	0	0
**Right Forelimb Wrist**		
Synovitis	0	3 (1.3)
Fibrosis	0	4 (2.0)
**Left Forelimb Elbow**		
Synovitis	0	0
Fibrosis	0	1 (2.0)
**Left Forelimb Wrist**		
Synovitis	0	4 (1.5)
Fibrosis	0	5 (2.0)

B

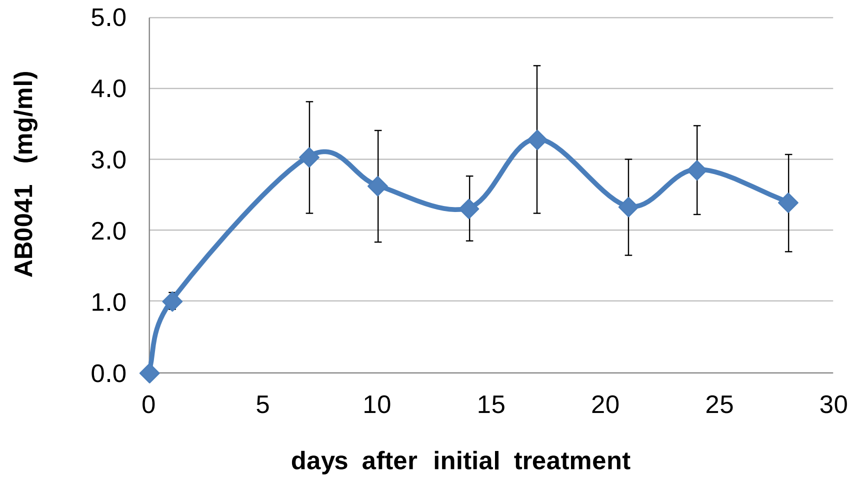

C

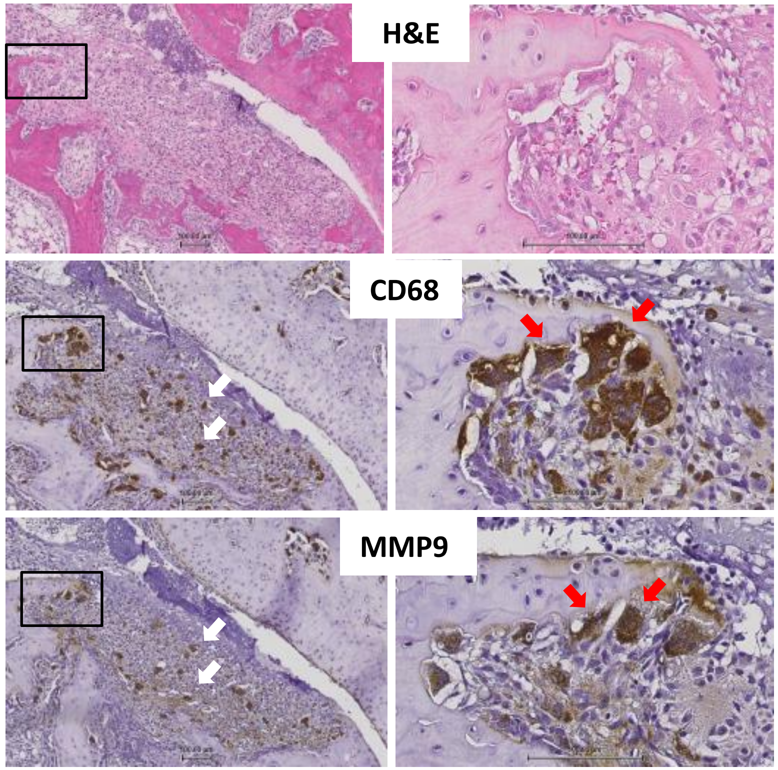

D

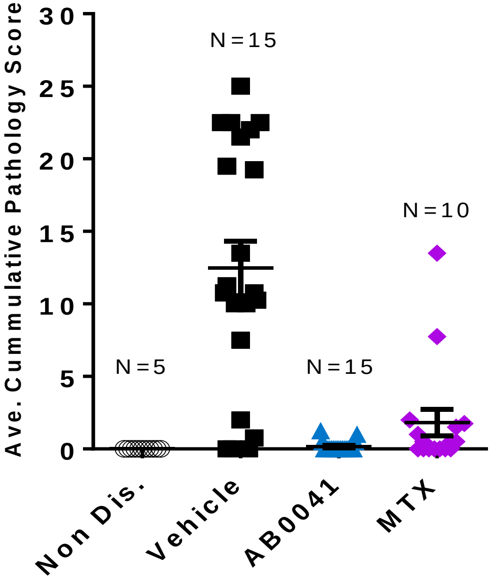

Supplement: S2 Fig — (A) Summary of histopathology analysis of limbs from the MSS study, showing no disease in AB0041-treated animals and mild to moderate synovitis and fibrosis in marimastat-treated animals. (B) Serum AB0041 titers during the course of the MSS study, demonstrate sustained exposure of > 2 mg/ml. (C) H&E-stained sections, and IHC for CD68 (macrophage and osteoclast marker) and MMP9 in rat CIA hind limbs. Images from serial sections at 100x (left) and 400x (right) magnification show a diseased joint with MMP9 expression in macrophages in pannus tissue (white arrows) and osteoclasts on the surface of the eroding bone (red arrows). (D) Histopathology analysis of rat CIA hind limbs. AB0041 treatment (50 mg/kg, twice weekly) reduced limb pathology to levels similar to those of healthy controls, and showed equivalent efficacy to the reference agent methotrexate (MTX). (PDF) [file pone.0127063.s003.pdf]
