# Supplementary figures and images for: Selective Allosteric Inhibition of MMP9 Is Efficacious in Preclinical Models of Ulcerative Colitis and Colorectal Cancer
Source: PLoS One. 2015 May 11;10(5):e0127063. doi: 10.1371/journal.pone.0127063 (PMC4427291; doi:10.1371/journal.pone.0127063)

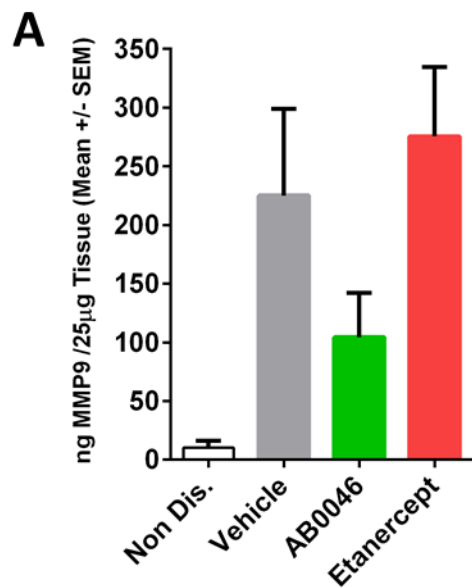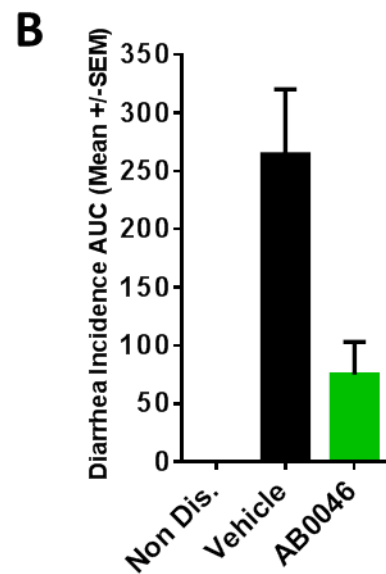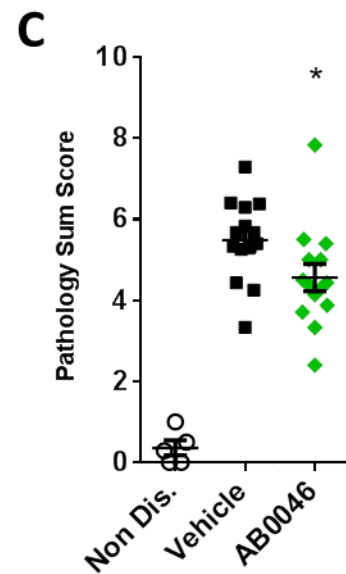

Supplement: S3 Fig — (A) MMP9 levels in mouse colon tissue were measured by ELISA. (B) The incidence of diarrhea was recorded and the AUC calculation was performed. (C) Blinded histopathological analysis was performed on colons excised at study termination. The degree of inflammation (primarily macrophages and neutrophils), of edema, and of necrosis was scored and a total pathology sum score was calculated. Statistical significance was assessed by one-way ANOVA with Dunnett’s Multiple Comparison post test (therapeutic study histopathology) or a Mann-Whitney test (prophylactic study histopathology). P value designations are as follows: * < 0.05, ** < 0.01, *** <0.001, **** < 0.0001. (PDF) [file pone.0127063.s004.pdf]
