# Supplementary material for: Selective Allosteric Inhibition of MMP9 Is Efficacious in Preclinical Models of Ulcerative Colitis and Colorectal Cancer
Source: PLoS One. 2015 May 11;10(5):e0127063. doi: 10.1371/journal.pone.0127063 (PMC4427291; doi:10.1371/journal.pone.0127063)
Supplement: S1 Methods — This section includes information supporting materials and methods for the following: histopathology and titer analyses for the rat MSS model; study design, histopathology, and IHC for the rat CIA study; ELISA analysis and prophylactic study design for the mouse DSS-induced colitis model. (PDF) [file pone.0127063.s005.pdf]

SUPPLEMENTARY MATERIALS AND METHODS

Rat MSS model

Histopathology

Hind limbs harvested from animals treated with AB0041, Vehicle, or Marimastat were fixed in 10% formalin. Limbs were then sent to IDEXX Laboratories (Fremont, CA) where they were processed into paraffin blocks, stained with hematoxylin and eosin (H&E), and examined microscopically.
The observed microscopic changes were graded utilizing a standard grading system whereby 0 = no significant change as compared to Vehicle tissue, 1 = minimal change, 2 = mild change, 3 = moderate change, and 4 = severe change. Limb joint components were evaluated for four soft-tissue changes and six bone/joint changes; see Table 2 for the specific parameters assessed in the histopathological evaluation.

AB0041 titer analysis

Levels of AB0041 in rat serum were measured by a direct binding enzyme-linked immunosorbent assay (ELISA). ELISA plates were coated with 2 μg/ml of human MMP9 (Gilead Biosciences, Foster City, CA) in 50 mM sodium borate overnight at 4 °C. The next day, plates were blocked with 5% BSA in PBS, pH 7.4 and washed with 0.05% Tween 20 in PBS (PBST). A standard curve of AB0041 was prepared by serially diluting AB0041 two-fold in PBST to generate a series ranging from 3000 ng/ml to 1.5 ng/ml. Serum samples were diluted at least 1:100 in PBST, and both standards and samples were then added to the pre-coated ELISA plate. After a one hour incubation, the plates were washed again and polyclonal goat-anti mouse IgG-HRP detection antibody (Thermo Scientific, Fair Lawn, NJ) was added to the plate at 1:10,000 dilution in 0.5% BSA/PBS. The plates were washed and signal was detected by addition of 3,3,5,5'-tetramethylbenzidine (TMB) (Sigma Aldrich, St. Louis, MO) for 2 minutes. The reaction was stopped by addition of 1M hydrochloric acid (HCl) and absorbance at 450 nm was measured on a Molecular Devices (Sunnyvale, CA) plate reader. AB0041 levels in serum were back-calculated using a four-parameter curve fit in the SoftMax software package (Molecular Devices).

Rat collagen-induced arthritis (CIA) study

Study design

The in vivo portion of the study was conducted at Invitek, Inc. (Hayward, CA). CIA was induced in male Lewis rats by intradermal injection at the base of the tail of 200 μg of bovine type II collagen (CII)

emulsified in Incomplete Freund's Adjuvant (IFA). Seven days after primary immunization, rats were given an intradermal booster injection of 100 µg CII in IFA. After the onset of disease (day seventeen), rats were randomized and assigned to different treatment groups to achieve an average clinical score (scoring system to qualitatively assess paw swelling and redness) per group of approximately two. Treatment groups, group size, route of administration, dose, dose volume, and dose frequency are summarized in Table 1.

Table 1. Study Design

Group	N =	ROA[a]	Dose (mg/kg)	Dose Frequency
Naïve (non-immunized)	5	IP	N/A	2x/week
Vehicle	15	IV	N/A	2x/week
AB0041	15	IV	50	2x/week
Methotrexate	10	PO	0.5	3x/week

a ROA = route of administration, IP = intraperitoneal, IV = intravenous, PO = per os (oral)

This study was carried out in strict accordance with the recommendations in the Guide for the Care and Use of Laboratory Animals of the National Institutes of Health.

Histopathology analysis

At the end of the study, rats were euthanized by CO_2 asphyxiation and hind limbs were removed. Small superficial incisions were made in the skin and the limb was placed in a 10x volume of 10% neutral buffered formalin, and transferred to IDEXX Laboratories (Sacramento, CA) for tissue processing, sectioning, and histopathology analysis. Hind limbs were trimmed and decalcified with ImmunocalTM (Decal Chemical Corporation) and then embedded in paraffin to enable longitudinal sections of each hind limb. Five micrometer sections were cut from paraffin blocks and stained with hematoxylin and eosin (H&E). Each limb was examined microscopically by a board certified veterinary pathologist for soft-tissue changes (edema, tissue/vessel necrosis, inflammatory cell infiltration, and fibroplasias) and cartilage and bone changes (cartilage erosion, bone erosion, periosteal bone formation, synovitis, pannus formation, and joint destruction) utilizing a standard severity score whereby: 0 = no significant change, 1 = minimal, 2 = mild, 3 = moderate and 4 = severe. The severity scores were based on global changes in each limb.

Immunohistochemistry analysis

The anti-MMP9 (ab76003)and anti-CD68 (ab125212)antibodies were obtained from Abcam (Cambridge, MA). All immunohistochemistry (IHC) reagents were from Biocare Medical (Concord, CA), unless

otherwise noted. Sections were cut from FFPE blocks at 5 micrometer thickness, placed on slides, and dried overnight. The slides were baked at 37°C overnight and then deparaffinized in two changes of xylene, a graded series of ethanol, then running water for 5 minutes each. After 30 minutes in Decal Retrieval Solution (MMP9 antibody) or Proteinase K Solution (CD68 antibody), the slides were placed in two changes each of 100% methanol, 70% methanol, and phosphate buffered saline (PBS) (5 minutes per change). The sections were then permeabilized in 0.3% Triton X-100 in PBS for 10 minutes after which they were washed in PBS. After blocking in Peroxidazed-1 for 20 minutes and Background Sniper for 30 minutes, the sections were incubated in primary antibody in Da Vinci Green Diluent for 30 minutes each. The sections were then incubated in MACH 2 HRP secondary antibody for 30 minutes and immunoreactivity was visualized with diaminobenzidine (DAB) chromagen. The slides were then counterstained, dehydrated in a graded series of ethanol, cleared in two changes of xylene and mounted using resin-based entellen (Electron Microscopy Sciences, Hatfield, PA). All slides were visualized and photographed using a Leica microscope.

Mouse DSS-induced colitis

Histopathology analysis

See main text Materials & Methods section.

Quantification of MMP9 in terminal colon tissue

A 1 cm section of frozen colon tissue from each mouse was homogenized in T-Per lysis buffer (Pierce, Rockford, IL) containing protease inhibitors (EDTA-free Mini Complete tablet, Roche, Indianapolis, IL) on ice with a hand-held electric homogenizer (Polytron 1300D, Kinematica, Lucerne, Switzerland). Protein levels were quantified using the BCA Protein Assay Kit (Pierce), and ELISA analysis for MMP9 (Mouse Total MMP9 Quantikine kit, R&D Systems) was conducted with 25 µg of protein from each sample. Total MMP9 levels were interpolated from a standard curve.

Prophylactic Study

In a 14 day prophylactic study, disease was established and scored as described in the main text Materials & Methods, and either PBST vehicle or AB0046 (30 mg/kg) were intraperitoneally administered on days -1, 2, 6, 9, and 12.
